# Systemic inflammatory biomarkers in relation to lung function and exercise‐induced bronchoconstriction in adolescents

**DOI:** 10.1111/pai.70231

**Published:** 2025-10-24

**Authors:** Karin Ersson, Kjell Alving, Margareta Emtner, Christer Janson, Henrik Johansson, Andrei Malinovschi

**Affiliations:** ^1^ Department of Medical Sciences, Clinical Physiology Uppsala University Uppsala Sweden; ^2^ Department of Women's and Children's Health, Physiotherapy Uppsala University Uppsala Sweden; ^3^ Department of Women's and Children's Health, Paediatrics Uppsala University Uppsala Sweden; ^4^ Department of Medical Sciences, Respiratory, Allergy and Sleep Research Uppsala University Uppsala Sweden

**Keywords:** adolescents, epidemiology, exercise‐induced bronchoconstriction, inflammatory biomarkers, proteomics, pulmonary function testing

## Abstract

**Introduction:**

The forced oscillation technique (FOT) complements spirometry in assessing lung function, with higher sensitivity to small airway dysfunction. Systemic inflammation is thought to influence lung development and exercise‐induced bronchoconstriction (EIB), but its relationship to circulating inflammatory proteins in adolescents is unclear.

**Objective:**

To investigate associations between systemic inflammatory biomarkers and baseline lung function and post‐exercise airway responses in adolescents.

**Methods:**

In 143 adolescents (13–15 years) from a population‐based cohort, baseline spirometry, FOT, and baseline blood samples were obtained. Participants completed an exercise challenge to assess EIB via changes in forced expiratory volume in 1 s (FEV_1_), resistance at 5 Hz (R_5_), and reactance at 5 Hz (X_5_). Plasma protein levels were measured using the proximity extension assay technique (Olink Target Inflammation and Immune Response panels). Associations with lung function (FEV_1_% predicted, R5, and X5 z‐scores) and post‐exercise responses (∆FEV_1_, ∆R5, ∆X5) were analyzed using linear regression with false discovery rate correction. Interaction with atopy was also examined.

**Results:**

Higher plasma levels of C‐C motif chemokine 19 (CCL19) were significantly associated with lower FEV_1_% predicted and lower X_5_ z‐scores at baseline, indicating reduced lung function and impaired small airway function. No proteins were associated with post‐exercise airway responses after correction. Five proteins showed significant interactions with atopy in relation to EIB.

**Conclusion:**

Elevated CCL19 may reflect systemic inflammatory processes contributing to impaired lung function in early adolescence. The observed atopy‐related interactions suggest the need to consider atopy in studies of systemic inflammation and airway physiology.

**Key message**

Elevated levels of the inflammatory protein CCL19 are associated with reduced baseline lung function and small airway impairment in adolescents, highlighting a potential role of systemic inflammation in early respiratory health; atopy may further modify these associations, emphasizing the importance of considering allergic status in future research on airway physiology.

## 1 INTRODUCTION

Lung function is usually measured by spirometry^1^ but the forced oscillation technique (FOT) is an alternative method used to evaluate the mechanical properties of the respiratory system during normal breathing. It works by superimposing small oscillations onto a person's spontaneous breathing to measure respiratory impedance.^2^ Oscillometric measurements can be especially useful for detecting lung function abnormalities in individuals with normal spirometry results, as oscillometry is reported to be more sensitive in detecting small airway dysfunction.^3^ Additionally, the correlation between self‐reported airway symptoms and oscillometric results tends to be stronger than that seen with spirometry, both in asthma patients^4^ and in the general population.^5^ Oscillometric parameters have also been shown to be sensitive in detecting airway hyperresponsiveness (AHR), both in asthmatic children^6^ and adults.^7^ AHR, characterized by an exaggerated bronchoconstrictive response to a standardized bronchial challenge, is both a marker of asthma severity and a predictor of future lung function impairment.^8^ To detect AHR, various types of indirect bronchial challenges with pre‐ and repeated post‐challenge lung function measurement can be employed, such as exercise, hyperpnea of dry air, or inhalation of hyperosmolar agents.^9^ In pediatric populations, exercise is the most adopted modality for bronchial challenge.^10^ Exercise‐induced bronchoconstriction (EIB) constitutes a distinct clinical manifestation of AHR, precipitated specifically by physical exertion.^11^

Lung function development can follow distinct trajectories from early life through adulthood and is influenced by both genetic and environmental factors. While most individuals reach normal peak lung function in early adulthood, a significant subset deviates from this path, exhibiting persistently low or declining lung function.^12^, ^13^ Persistently low lung function in adolescence is clinically important, as it has been linked to a heightened risk of developing chronic obstructive pulmonary disease (COPD) or asthma in later life.^12^, ^14^

Inflammation is very likely to be a factor with regard to lung function development. In individuals with persistent childhood asthma, elevated blood eosinophil counts (BEC) have been linked to attenuated lung growth and premature decline in pulmonary function.^15^ This inflammatory burden may disrupt airway remodeling processes, potentially resulting in fixed airflow limitation later in life.^14^ Evidence suggests that individuals with asthma who exhibit AHR and eosinophilic inflammation in adolescence are more likely to follow adverse lung function trajectories.^8^, ^16^ Enhancing our understanding of the inflammatory mechanisms underlying both abnormal lung function and airway hyperresponsiveness in adolescence is essential for early identification of at‐risk individuals and for the development of targeted interventions.

Multiplex proteomic assays offer the possibility of broader analyses of the inflammatory profile by allowing the simultaneous measurement of numerous protein biomarkers that may be relevant to lung function outcomes. Previous studies have investigated the relationship between proteomics and lung function in adults^17^, ^18^, ^19^, ^20^; however, similar research in adolescent populations remains limited.

We aimed to explore the relationship between systemic inflammatory biomarkers, assessed by a sensitive multiarray technique, and lung function as well as airway hyperresponsiveness measured by spirometry and oscillometry in a population‐based sample of adolescents.

## 2 METHODS

**FIGURE 1:**
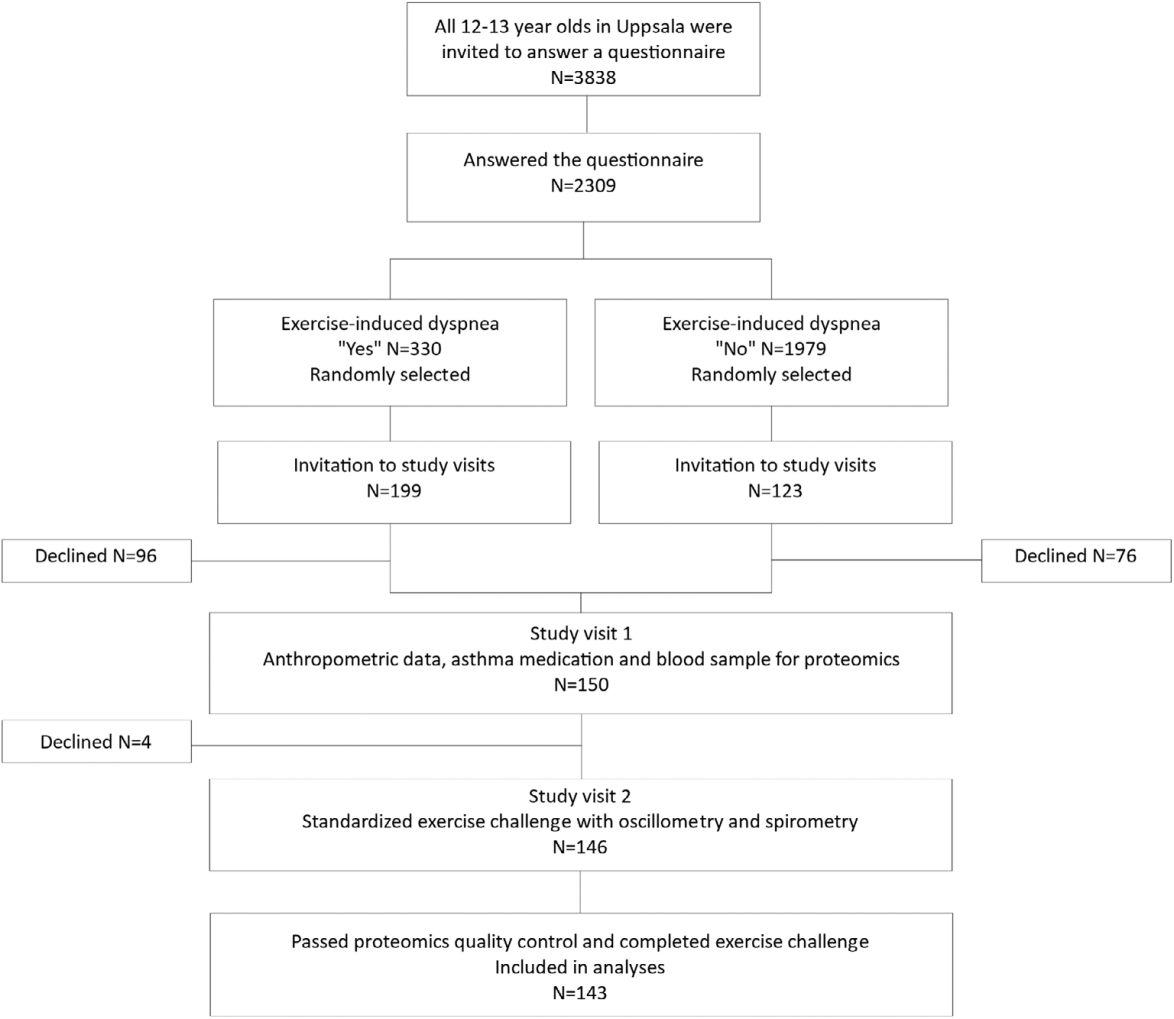
Study inclusion.

Participants were recruited from a population‐based study of the prevalence of exercise‐induced bronchoconstriction (EIB) in adolescents (age 13–15).^21^ Study inclusion is presented in Figure 1. The study included 2309 adolescents who answered a questionnaire about exercise‐induced respiratory problems. After stratification of the adolescents according to their answer of yes or no to the question of having experienced exercise‐induced dyspnea in the last 12 months, 150 participants were randomly selected and invited to two study visits. During the first visit, anthropometric data, information on asthma medication, and venous blood samples for analyses of IgE sensitization and plasma proteins were collected.

Atopic status was assessed by IgE against a mix of aeroallergens (mite, furry animals, mold, grass, tree, and weed pollen) (Phadiatop; ImmunoCAP; Thermo Fisher Scientific, Uppsala, Sweden). Participants with IgE levels ≥0.1 kU_A_/L were categorized as atopic.^22^

During the second visit AHR was assessed by a standardized exercise challenge investigating EIB. Time between the two visits were median 12 days (interquartile range 6–19 days).

### 2.1 Exercise challenge

The participants were instructed to refrain from vigorous physical activity, caffeine, nicotine, and heavy meals for 4 h prior to the test. Additionally, they were advised not to use any short‐acting β2‐agonists for 8 h, long‐acting β2‐agonists for 24, and leukotriene receptor antagonists for 72 h prior to the test, as well as no inhaled corticosteroids on the day of the test.

Before the treadmill exercise challenge, baseline lung function was assessed using FOT (Resmon Pro Full, Restech Srl) and spirometry (CardioPerfect dynamic spirometry; Welch Allyn). FOT was measured in duplicate using a multifrequency signal at 5, 11, and 19 Hz. Baseline respiratory resistance at 5 Hz (R_5_) and respiratory reactance at 5 Hz (X_5_) were calculated as the mean of two acceptable measurements, each consisting of 10 valid breaths. Spirometry followed the ATS/ERS guidelines, and forced expiratory volume in 1 s (FEV_1_) was recorded as the highest of three acceptable and reproducible maneuvers, with the two highest values differing by no more than 150 mL.^23^

During the exercise, participants wore a nose clip and breathed dry air (H_2_O <5 mg/L, 18°C–22°C) through a tubing system connected to a central gas container. Heart rate was continuously monitored, and the protocol required participants to reach 90% of their predicted maximum heart rate ((220 – age) ×0.9) within the first 2 min, maintaining this heart rate during 5–6 min. Post‐exercise, FOT (with at least five artifact‐free breaths) and spirometry (duplicate measurements) were performed at 5, 10, 15, and 30 min after exercise cessation. Participants were defined as EIB‐positive if ∆FEV_1_ ≤−10%.

### 2.2 Proteomics analyses

Two high‐throughput multiplex immunoassays were employed, each measuring 92 protein biomarkers simultaneously. The proteins were pre‐selected by the manufacturer in two standard panels: the Olink Target 96 Inflammation and Immune Response panels (SciLifeLab, Uppsala, Sweden).^24^ All analyzed proteins are presented in Tables S1. Four proteins are present in both employed panels; these proteins were included from the Inflammation panel only. Out of the 180 proteins, 45 proteins (19 Inflammation, 26 Immune response) with more than 25% of the samples below the limit of detection (LOD) were excluded from the analyses. In the remaining 135 proteins, the actual values below LOD were used in the analyses. The results are given as a log2‐transformed relative quantification unit: normalized protein expression (NPX). In total, 143 out of 150 participants passed the manufacturer's quality control for both panels and completed the exercise challenge and were thus included in the analyses. Pathway analyses of relevant proteins were performed using Olink Insight.

Data collection from questionnaires and exercise challenges was performed in 2012–2013. Proteomics analyses were done in stored samples as an add‐on study in 2023–2024.

### 2.3 Statistical analysis

Normality of continuous variables was evaluated using the Shapiro–Wilk test. Variables that followed a normal distribution were reported as means with standard deviations (SD), while those that did not were summarized as medians with interquartile ranges (Q1–Q3).

Airway responses after exercise challenge were defined as follows: ∆FEV_1_ as the percentage change from baseline to the lowest value after exercise; ∆R5 as the percentage change from baseline to the peak value post‐exercise; and ∆X5 as the absolute change from baseline to the lowest post‐exercise value.

Linear regression models providing β‐coefficients and 95% CIs were used to examine associations between levels of plasma proteins (as exposures) and baseline lung function: FEV_1_ as percent predicted, R_5_ as z‐score, and X_5_ as z‐score (as outcomes). For the outcome FEV_1_% predicted, crude models were used, while for outcomes R_5_ z‐scores and X_5_ z‐score, the models were adjusted for sex. Linear regression models were also employed to investigate associations between levels of plasma proteins (as exposures) and airway responses after exercise challenge ∆FEV_1_, ∆R_5_, and ∆X_5_ (as outcomes). These models were adjusted for sex. To account for multiple testing, a false discovery rate (FDR) of 0.05 was applied using the Benjamini Hochberg (BH) procedure.

In secondary analyses, the effects of atopic status on associations between proteins and airway responses after exercise were assessed. This was done by the same models as the primary analysis described above, but with the addition of an interaction term protein × atopy.

A *p*‐value <.05 was considered statistically significant. All statistical analyses were conducted using STATA version 16.1 (StataCorp LLC, College Station, TX).

### 2.4 Ethics

This study was approved by the Ethics Review Board in Uppsala, Sweden (Dnr 2011/413). All participants and their guardians provided informed written consent.

## 3 RESULTS

Out of 143 participants (female *N* = 87), 48 (33.6%) were EIB positive by exercise challenge. Atopy was found in 84 (59%) participants. Characteristics of the study participants are presented in Table 1.

**TABLE 1:** Characteristics of the study participants, presented for the whole population and stratified according to atopy.

	All participants	Non atopic	Atopic
Number of participants	143	59	84
Female, *n* (%)	87 (60.8)	42 (71.2)	45 (53.6)
Age (years), median (Q1‐Q3)	14 (14–15)	14 (14–15)	14 (14–15)
Height (cm), median (Q1‐Q3)	168 (162–175)	166 (161–172)	170 (164–176)
BMI categories^a^, *n* (%)			
Overweight >+1 SD Normal −2SD – +1SD Thinness <−2 SD	24 (16.8) 108 (75.5) 6 (4.2)	8 (13.6) 47 (79.7) 0	16 (19.1) 61 (72.6) 6 (7.1)
Rhinitis, *n* (%)	48 (33.6)	8 (13.6)	40 (47.6)
Current asthma^b^, *n* (%)	43 (30)	15 (25.4)	28 (33.3)
Wheeze^c^, *n* (%)	56 (39.2)	21 (35.6)	35 (42.2)
ICS^d^, *n* (%)	23 (16.1)	5 (8.5)	18 (21.4)
FEV_1_% predicted^e^, mean (SD)	93.7 (±10.3)	94.3 (±10.5)	93.4 (±10.1)
Abnormal FEV_1_/FVC ratio^f^, *n* (%)	10 (7)	7 (11.9)	3 (3.6)
R_5_ (cmH_2_0*s/L), mean (SD)	3.6 (±0.77)	3.75 (±0.56)	3.49 (±0.62)
Abnormal R_5_ ^g^, *n* (%)	25 (17.5)	11 (18.6)	14 (16.3)
X_5_ (cmH_2_0*s/L), mean (SD)	−1.04 (±0.44)	−1.09 (±0.23)	−0.98 (±0.25)
Abnormal X_5_ ^h^, *n* (%)	10 (7)	4 (6.8)	6 (7.1)
EIB positive^i^, *n* (%)	48 (33.6)	19 (32.2)	29 (34.5)
∆FEV_1_, median (min, max)	−6.3 (−41.9, 5.8)	−5.6 (−25.5, 4.3)	−6.5 (−41.9, 5.8)
∆R5, median (min, max)	21.9 (−8.9, 142.4)	23.2 (−4.8, 66.9)	21.4 (−8.9, 142.4)

Abbreviations: BMI, body mass index; CI, confidence interval; EIB, exercise‐induced bronchoconstriction; FEV_1_, forced expiratory volume in one second; ICS, inhaled corticosteroid; Q1, first quartile; Q3, third quartile; R_5_, respiratory resistance at 5 Hz; SD, standard deviation; X_5_, respiratory reactance at 5 Hz.

^a^ Cutoffs according to World Health Organization.

^b^ Self‐reported physician‐diagnosed with symptoms and/or medication.

^c^ Self‐reported symptom in the preceding 12 months.

^d^ Any use in the preceding 3 months.

^e^ Reference value: Global Lung Initiative.

^f^ <, Lower limit of normal according to Global Lung Initiative.

^g^ >, Upper limit of normal according to the reference equation by Ducharme.

^h^ <, Lower limit of normal according to the reference equation by Ducharme.

^i^ EIB defined by spirometry as ΔFEV1 ≤−10%.

Out of the 135 studied proteins, the level C‐C motif chemokine 19 (CCL19) associated with baseline lung function after BH correction with FDR set at 0.05. CCL19 was negatively associated with FEV_1_% predicted and X5 z‐score. There were no significant associations between any protein and R_5_ z‐score, although tumor necrosis factor (TNF) and CCL19 had a trend for positive association with R_5_ (*p* < .10) (Table 2). In Table 2, all proteins with significant associations for each outcome before BH correction are included.

**TABLE 2:** Associations between levels of plasma proteins and baseline lung function measurements analyzed by linear regression models and presented per outcome.

		Estimates	95% CI	*q*‐value
FEV_1_%				
CCL19	C‐C motif chemokine 19	−4.96	(−7.57, −2.35)	.037
LIFR	Leukemia inhibitory factor receptor	−9.95	(−18.18, −1.73)	.952
CD8A	T‐cell surface glycoprotein CD8 alpha chain	3.61	(0.47, 6.75)	.952
IFNLR1	Interferon lambda receptor 1	−5.22	(−9.99, −0.45)	.952
IL17C	Interleukin‐17C	2.44	(0,17, 4.71)	.952
CNTNAP2	Contactin‐associated protein‐like 2	−3.95	(−7.84, −0.06)	.952
R_5_ z‐score				
TNF	Tumor necrosis factor	0.64	(0.29, 0.98)	.054
CCL19	C‐C motif chemokine 19	0.41	(0.18, 0.65)	.062
TREM1	Triggering receptor expressed on myeloid cells 1	0.62	(0.05, 1.18)	.999
X_5_ z‐score				
CCL19	C‐C motif chemokine 19	−0.53	(−0.82, −0.25)	.048
PTH1R	Parathyroid hormone 1 receptor	0.53	(0.04, 1.01)	.908
BTN3A2	Butyrophilin subfamily 3 member A2	0.64	(0.05, 1.23)	.908
CXADR	Coxsackievirus and adenovirus receptor	0.60	(0.04, 1.16)	.908

*Note*: For FEV_1_% crude models were used and for R_5_ z‐score and X_5_ z‐scores the models were adjusted for sex. *q*‐values were derived by Benjamini Hochberg correction with false discovery rate .05. All proteins with significant associations for each outcome before BH correction are included in the table.

Abbreviations: CI, confidence interval; FEV_1_, forced expiratory volume in one second; R_5_, respiratory resistance at 5 Hz; X_5_, respiratory reactance at 5 Hz.

Pathway analyses indicate that CCL19 contributes to immune responses and lymphocyte circulation, while TNF is implicated in inflammation and the regulation of cell proliferation, differentiation, and immune signaling.

No proteins were significantly associated with airway response after exercise challenge measured by any of the outcomes (∆FEV_1_, ∆R_5_ or ∆X_5_) when BH correction with FDR set at 0.05 was used (Table 3). In Table 3, all proteins with significant associations for each outcome before BH correction are included.

**TABLE 3:** Associations between levels of plasma proteins and airway response after exercise challenge analyzed by linear regression models and presented per outcome.

		Estimates	95% CI	*q*‐Value
∆FEV_1_				
FGF21	Fibroblast growth factor 21	−1.38	(0.32, 2.43)	.967
IL15RA	Interleukin‐15 receptor subunit alpha	−6.00	(0.28, 11.71)	.967
CD8A	T‐cell surface glycoprotein CD8 alpha chain	−2.59	(0.08, 5.10)	.967
∆R_5_				
IL15RA	Interleukin‐15 receptor subunit alpha	32.71	(13.32, 52.09)	.163
IL10RB	Interleukin‐10 receptor subunit beta	23.94	(6.13, 41.74)	.635
GLB1	Beta‐galactosidase	−10.54	(−19.54, −1.54)	.653
DPP10	Inactive dipeptidyl peptidase 10	−17.10	(−31.76, −2.45)	.653
FGF19	Fibroblast growth factor 19	5.70	(0.80, 10.60)	.653
ITGB6	Integrin beta‐6	−15.72	(−29.67, −1.76)	.654
CLEC4C	C‐type lectin domain family 4 member C	−11.36	(−22.21, −0.51)	.697
STC1	Stanniocalcin‐1	−12.58	(−24.79, −0.38)	.697
CCL19	C‐C motif chemokine 19	−7.85	(−15.50, −0.20)	.697
∆X_5_				
FGF19	Fibroblast growth factor 19	−0.14	(−0.26, −0.02)	.979
ITM2A	Integral membrane protein 2A	0.15	(0.01, 0.30)	.979

*Note*: Presented per outcome. For all outcomes the models were adjusted for sex. *q*‐values were derived by Benjamini Hochberg correction with false discovery rate .05. All proteins with significant associations for each outcome before BH correction are included in the table.

Abbreviations: CI, confidence interval; FEV_1_, forced expiratory volume in one second; R_5_, respiratory resistance at 5 Hz; X_5_, respiratory reactance at 5 Hz.

**FIGURE 2:**
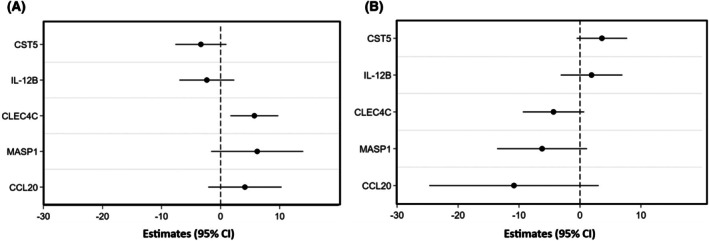
Associations between levels of plasma proteins and airway response after exercise challenge in analyses stratified by atopy, using linear regression models adjusted for sex. The direction of association is shown on the *x*‐axis (negative to the left and positive to the right). Non‐atopic in panel (A) and atopic in panel (B). Only proteins with significant interaction term protein × atopy in relation to airway response after exercise challenge were analyzed. In the models with CST5, IL12B, CLEC4C, and MASP1 the outcome was ∆FEV_1_, while for CCL20 the outcome was ∆R_5_. CST5, Cystatin‐D; IL‐12B, Interleukin‐12 receptor subunit beta‐1; CLEC4C, C‐type lectin domain family 4 member; MASP1, Mannan‐binding lectin serine protease 1; CCL20, C‐C motif chemokine 20; CI, confidence interval; FEV_1_, forced expiratory volume in one second; R_5_, respiratory resistance at 5 Hz.

In secondary analyses, we studied possible interaction with atopy on the relation between plasma proteins and airway response after exercise challenge. We found four significant interactions for ∆FEV_1_: Cystatin‐D, CST5; Interleukin‐12 receptor subunit beta‐1, IL12B; C‐type lectin domain family 4 member, CLEC4C; Mannan‐binding lectin serine protease 1, MASP1; and one interaction term for ∆R_5_: C‐C motif chemokine 20, CCL20. For associations from stratified analyses, see Figure 2. No interaction was identified for any of the models with the outcome ∆X_5_.

In pathway analyses, these proteins were identified as regulators of immunity and immune modulation: CLEC4C facilitates antigen uptake and signaling in dendritic cells; MASP1 promotes complement activation in innate defense; CST5 inhibits cysteine proteases to protect tissues; IL12B forms cytokines that stimulate immune cells and drive inflammation; and CCL20 recruits immune cells, enhances mucosal defense, and exhibits antimicrobial effects. Together, they are involved in pathogen recognition, immune activation, regulation, and tissue protection.

## 4 DISCUSSION

In this population‐based study of adolescents, we investigated associations between systemic inflammatory and immune regulatory biomarkers and lung function—both at baseline and following a standardized exercise challenge—using spirometry and oscillometry. Among the 135 plasma proteins assessed, we identified C‐C motif chemokine 19 (CCL19) as significantly associated with baseline lung function after correction for multiple testing. Specifically, higher levels of CCL19 were associated with lower FEV_1_% predicted and X_5_ z‐scores, suggesting a potential role for this chemokine in abnormal airway mechanics. However, we found no significant associations between circulating protein biomarkers and airway responses after exercise challenge in the overall cohort. Notably, in stratified analyses, atopy modified the relationship between several plasma proteins and airway responses, indicating that systemic inflammation may differentially influence AHR in atopic versus non‐atopic adolescents.

CCL19 is a homeostatic chemokine involved in the migration of dendritic cells and T lymphocytes to lymphoid tissues, where it contributes to immune surveillance and inflammatory responses.^25^ Our finding that CCL19 correlates negatively with both FEV_1_% predicted and X_5_ z‐scores, reflecting impaired elastic recoil or increased peripheral airway stiffness, implies that CCL19 may play a role in airway dysfunction. In line with our findings, Wang et al. have found an association between higher levels of circulating CCL19 and having low lung function in young adults, at the age of 24 years.^19^ Elevated levels of CCL19 have previously been associated with allergic airway inflammation and asthma in murine models^26^, ^27^ and in human tissue.^28^ Recent mechanistic evidence further supports that CCL19 actively promotes Th2 cell differentiation, exacerbating type 2 immune responses and worsening allergic airway inflammation.^29^ These immune‐mediated effects can contribute to abnormal lung function outcomes.

In addition to CCL19, tumor necrosis factor (TNF) demonstrated a trend towards a positive association with R_5_ z‐scores. This aligns with the current knowledge of TNF as a key mediator of airway inflammation and remodeling.^30^ TNF has been shown to increase airway smooth muscle tone, mucus production, and epithelial permeability—all processes that can contribute to increased airway resistance.^31^ Elevated TNF levels have also been linked to refractory asthma phenotypes, particularly in cases with prominent small airway involvement.^32^ Anti‐TNF treatment resulted in decreased AHR and improved lung function in refractory asthma.^32^ The observed trend between TNF and R_5_ in our adolescent population may therefore reflect subclinical inflammatory processes influencing airway resistance and warrants further investigation in larger cohorts.

No individual protein biomarker was significantly associated with airway response following exercise when applying stringent false discovery rate correction. This may reflect the fact that the process is dynamic and EIB results in an increase of mast cell mediators after an exercise challenge. Most of the studies demonstrating relations between mast cell mediators and EIB often measured mast cell mediators in urine^33^ or induced sputum.^34^ This suggests the transient nature of exercise‐induced changes that might be difficult to capture using single pre‐challenge proteomic measurements. However, previous studies have shown elevated concentrations of mast cell–associated mediators in exhaled condensate among children with EIB,^35^ and baseline fractional exhaled nitric oxide (FeNO) has similarly been linked to its presence in the present population.^36^ In line with these findings, airway hyperresponsiveness to direct provocation in children has been associated with increased eosinophil activation, evidenced by higher levels of eosinophil‐derived neurotoxin and eosinophil cationic protein.^37^ These observations indicate that while single baseline proteomic measurements may lack sensitivity for detecting exercise‐induced responses, airway inflammatory profiles at rest may still provide important insights into the underlying susceptibility to EIB.

Interestingly, our interaction analysis revealed that associations between several plasma proteins with ∆FEV_1_ or ∆R_5_ were significantly different in atopic versus non‐atopic individuals. This includes CLEC4C, MASP1, CST5, IL12B, and CCL20. Since these proteins are linked to both innate and adaptive immune pathways, their divergent associations across groups may reflect distinct inflammatory endotypes contributing to airway responsiveness in EIB. This interpretation is consistent with previous evidence showing that airway inflammation and hyperresponsiveness vary by atopic background, with atopic individuals more likely to exhibit eosinophilic inflammation and IgE‐mediated responses.^38^, ^39^ Furthermore, studies using FeNO measurement support this link, as elevated levels have been associated with both atopy and heightened airway responsiveness in asthma.^40^

This study benefits from a population‐based design, the assessment of AHR through objective measurement of EIB by standardized exercise challenge, the simultaneous assessment of spirometric and oscillometric parameters, and use of established proteomics panels, reflecting inflammation and immune regulation processes. Nonetheless, several limitations should be acknowledged. First, the cross‐sectional design limits inference about the directionality of associations and therefore precludes causal conclusions regarding the observed relationships and interactions. Second, analyses of EIB could have been strengthened by a study design that included measurements of inflammatory proteins both before and after the exercise challenge. Third, our sample size, while substantial for an in‐depth clinical study, may have limited statistical power in the context of high‐dimensional proteomic data and interaction testing. Additionally, circulating plasma biomarkers may not fully reflect airway‐specific inflammation, particularly in mild or early‐stage disease. Furthermore, we acknowledge that this analysis is not fully unbiased as it relied on predefined protein panels and may have excluded other relevant proteins involved in airway inflammation and airway physiology. Lastly, the external validity of our findings is limited by the study population, which consisted of adolescents living in Sweden and primarily White, constraining transferability to adolescents in the global context.

## 5 CONCLUSION

Our findings indicate that higher levels of systemic CCL19 are associated with lower baseline lung function in adolescents, suggesting a potential link between circulating chemokine activity and small airway impairment. Although no plasma proteins were broadly associated with EIB outcomes, the observed interactions with atopy point to underlying immune variation in airway reactivity. These findings highlight the importance of considering atopic status in interpreting systemic inflammatory signals.

## AUTHOR CONTRIBUTIONS

**Karin Ersson:** Writing – original draft; writing – review and editing; methodology; formal analysis; visualization; investigation; data curation. **Kjell Alving:** Conceptualization; funding acquisition; writing – review and editing; methodology; investigation; resources. **Margareta Emtner:** Writing – review and editing. **Christer Janson:** Writing – review and editing. **Henrik Johansson:** Investigation; funding acquisition; writing – review and editing; methodology; supervision; conceptualization. **Andrei Malinovschi:** Conceptualization; investigation; funding acquisition; writing – review and editing; methodology; supervision; resources; formal analysis.

## FUNDING INFORMATION

This study was sponsored by the Swedish Research Council, the Swedish Heart and Lung Foundation, the Swedish Asthma and Allergy Association, the Knut and Alice Wallenberg Foundation, the Hesselman Foundation, the Signhild Engqvist Foundation, the Bror Hjerpstedt Foundation, the Gillbergska Foundation, and the Uppsala County Association against Heart and Lung Diseases.

## CONFLICT OF INTEREST STATEMENT

The authors declare no conflict of interest related to this work.

## PEER REVIEW

The peer review history for this article is available at https://www.webofscience.com/api/gateway/wos/peer‐review/10.1111/pai.70231.

## Supporting information


Table S1.
