# Supplementary material for: Systemic inflammatory biomarkers in relation to lung function and exercise‐induced bronchoconstriction in adolescents
Source: Pediatr Allergy Immunol. 2025 Oct 24;36(10):e70231. doi: 10.1111/pai.70231 (PMC12550650; doi:10.1111/pai.70231)
Supplement: Supplementary file 1 — Table S1. [file PAI-36-e70231-s001.zip › pai70231-sup-0001-TableS1-S3@Supplementary_material_baseline_lung_function.docx]

| **Table S1.** Associations between levels of plasma proteins and forced expiratory volume in one second (FEV_1_) as % predicted analyzed by crude linear regression models. P-values are uncorrected while q-values were derived by Benjamini Hochberg correction with false discovery rate 0.05. | | | | |
| --- | --- | --- | --- | --- |
| **Variable** | **Estimates** | **(95% CI)** | **p-value** | **q-value** |
| **CCL19** | **-4.96** | **(-7.567, -2.354)** | **0.0003** | **0.0373** |
| LIFR | -9.951 | (-18.176, -1.727) | 0.0191 | 0.9519 |
| CD8A | 3.608 | (0.468, 6.749) | 0.0259 | 0.9519 |
| IFNLR1 | -5.22 | (-9.991, -0.449) | 0.0337 | 0.9519 |
| IL17C | 2.438 | (0.171, 4.706) | 0.0368 | 0.9519 |
| CNTNAP2 | -3.951 | (-7.843, -0.059) | 0.0486 | 0.9519 |
| STC1 | -4.178 | (-8.378, 0.021) | 0.0531 | 0.9519 |
| CCL11 | 4.298 | (-0.132, 8.727) | 0.0592 | 0.9519 |
| KRT19 | 2.017 | (-0.107, 4.142) | 0.0648 | 0.9519 |
| CLEC4C | -3.281 | (-6.978, 0.417) | 0.0842 | 0.9519 |
| DPP10 | -4.35 | (-9.277, 0.576) | 0.0856 | 0.9519 |
| CD40 | 3.325 | (-0.476, 7.127) | 0.0886 | 0.9519 |
| HNMT | -2.615 | (-5.676, 0.446) | 0.0963 | 0.9519 |
| IL6 | -2.425 | (-5.32, 0.47) | 0.1028 | 0.9519 |
| EBP1 | 1.81 | (-0.434, 4.053) | 0.1161 | 0.9519 |
| CLEC4D | 2.513 | (-0.604, 5.63) | 0.1163 | 0.9519 |
| CDCP1 | -4.374 | (-9.943, 1.195) | 0.1259 | 0.9519 |
| PTH1R | -3.184 | (-7.265, 0.897) | 0.1284 | 0.9519 |
| MILR1 | -2.512 | (-5.829, 0.804) | 0.1398 | 0.9519 |
| CXCL10 | -1.565 | (-3.637, 0.507) | 0.1410 | 0.9519 |
| STAMBP | 1.383 | (-0.523, 3.289) | 0.1573 | 0.9579 |
| MASP1 | -4.239 | (-10.282, 1.804) | 0.1713 | 0.9579 |
| ITGB6 | 3.333 | (-1.466, 8.132) | 0.1756 | 0.9579 |
| TRIM21 | 1.542 | (-0.686, 3.77) | 0.1771 | 0.9579 |
| CCL20 | -1.821 | (-4.454, 0.812) | 0.1774 | 0.9579 |
| MCP4 | 1.988 | (-1.003, 4.979) | 0.1947 | 0.9732 |
| DCBLD2 | -3.342 | (-8.389, 1.705) | 0.1964 | 0.9732 |
| IL15RA | 4.685 | (-2.592, 11.963) | 0.2090 | 0.9732 |
| CASP8 | 2.697 | (-1.527, 6.921) | 0.2128 | 0.9732 |
| SCF | -3.648 | (-9.584. 2.287) | 0.2303 | 0.9732 |
| SIRT2 | 0.877 | (-0.607, 2.36) | 0.2488 | 0.9732 |
| IL18 | 2.39 | (-1.726, 6.506) | 0.2571 | 0.9732 |
| NT3 | 3.19 | (-2.353, 8.733) | 0.2612 | 0.9732 |
| CCL23 | 2.05 | (-1.562, 5.662) | 0.2679 | 0.9732 |
| IL8 | 2.249 | (-1.918, 6.416) | 0.2920 | 0.9732 |
| FGF21 | -0.725 | (-2.089, 0.638) | 0.2989 | 0.9732 |
| CXCL6 | 1.393 | (-1.258, 4.044) | 0.3047 | 0.9732 |
| LAMP3 | 1.936 | (-1.78, 5.653) | 0.3089 | 0.9732 |
| **Variable** | **Estimates** | **(95% CI)** | **p-value** | **q-value** |
| IL12B | -2.046 | (-5.978, 1.885) | 0.3094 | 0.9732 |
| CD83 | -2.454 | (-7.277, 2.368) | 0.3202 | 0.9732 |
| IL10 | -1.566 | (-4.864, 1.731) | 0.3533 | 0.9732 |
| AXIN1 | 0.574 | (-0.646, 1.793) | 0.3580 | 0.9732 |
| TWEAK | -2.676 | (-8.444, 3.091) | 0.3646 | 0.9732 |
| GLB1 | 1.449 | (-1.707, 4.605) | 0.3697 | 0.9732 |
| PRDX1 | 1.164 | (-1.385, 3.712) | 0.3723 | 0.9732 |
| OSM | 1.059 | (-1.335, 3.453) | 0.3873 | 0.9732 |
| MMP1 | 0.86 | (-1.094, 2.815) | 0.3898 | 0.9732 |
| MCP2 | 1.111 | (-1.476, 3.697) | 0.4013 | 0.9732 |
| CCL4 | 1.444 | (-1.991, 4.879) | 0.4113 | 0.9732 |
| FGF19 | -0.749 | (-2.555, 1.056) | 0.4174 | 0.9732 |
| OPG | -2.421 | (-8.279, 3.436) | 0.4192 | 0.9732 |
| IRF9 | -1.172 | (-4.065, 1.721) | 0.4285 | 0.9732 |
| CXCL1 | 0.736 | (-1.154, 2.625) | 0.4467 | 0.9732 |
| TRANCE | -1.071 | (-3.911, 1.768) | 0.4609 | 0.9732 |
| CKAP4 | -1.308 | (-4.919, 2.303) | 0.4790 | 0.9732 |
| GDNF | 1.253 | (-2.248, 4.754) | 0.4841 | 0.9732 |
| NCR1 | -1.494 | (-5.69, 2.702) | 0.4864 | 0.9732 |
| uPA | 1.919 | (-3.72, 7.558) | 0.5058 | 0.9732 |
| CCL3 | 0.852 | (-1.678, 3.382) | 0.5103 | 0.9732 |
| PDL1 | 1.344 | (-2.689, 5.377) | 0.5146 | 0.9732 |
| CXCL5 | -0.42 | (-1.715, .875) | 0.5256 | 0.9732 |
| ENRAGE | 0.918 | (-1.972, 3.807) | 0.5345 | 0.9732 |
| IL10RA | -0.584 | (-2.446, 1.279) | 0.5401 | 0.9732 |
| AREG | -0.748 | (-3.14, 1.644) | 0.5408 | 0.9732 |
| LAPTGFbeta1 | 1.438 | (-3.282, 6.158) | 0.5513 | 0.9732 |
| ADA | 1.291 | (-3.065, 5.647) | 0.5623 | 0.9732 |
| CX3CL1 | 1.402 | (-3.501, 6.304) | 0.5761 | 0.9732 |
| MCP1 | 1.312 | (-3.301, 5.925) | 0.5780 | 0.9732 |
| KLRD1 | -0.999 | (-4.523, 2.524) | 0.5792 | 0.9732 |
| CLEC6A | 0.979 | (-2.505, 4.464) | 0.5826 | 0.9732 |
| FGF2 | 0.506 | (-1.311, 2.323) | 0.5862 | 0.9732 |
| SLAMF1 | 1.387 | (-3.792, 6.567) | 0.6004 | 0.9732 |
| LY75 | -1.218 | (-6.01, 3.574) | 0.6192 | 0.9732 |
| VEGFA | 1.612 | (-4.891, 8.115) | 0.6278 | 0.9732 |
| CD6 | -1.103 | (-5.558, 3.351) | 0.6281 | 0.9732 |
| IFNgamma | -0.499 | (-2.532, 1.534) | 0.6311 | 0.9732 |
| DAPP1 | 0.2 | (-0.624, 1.024) | 0.6347 | 0.9732 |
| TNFRSF9 | -0.764 | (-3.928, 2.4) | 0.6368 | 0.9732 |
| NF2 | 0.333 | (-1.069. 1.736) | 0.6422 | 0.9732 |
| SH2B3 | 0.23 | (-0.768, 1.228) | 0.6518 | 0.9732 |
| ITGA11 | -0.73 | (-3.938, 2.479) | 0.6564 | 0.9732 |
| **Variable** | **Estimates** | **(95% CI)** | **p-value** | **q-value** |
| TNF | -0.906 | (-5.049, 3.237) | 0.6689 | 0.9732 |
| TREM1 | 0.964 | (-3.535, 5.462) | 0.6753 | 0.9732 |
| FGF23 | 0.625 | (-2.331, 3.58) | 0.6792 | 0.9732 |
| IL10RB | 1.284 | (-4.791, 7.359) | 0.6793 | 0.9732 |
| LAG3 | -1.008 | (-6.019, 4.003) | 0.6939 | 0.9732 |
| SIT1 | 0.684 | (-2.785, 4.154) | 0.6996 | 0.9732 |
| FAM3B | 0.669 | (-2.739, 4.077) | 0.7011 | 0.9732 |
| PIK3AP1 | 0.345 | (-1.483, 2.173) | 0.7119 | 0.9732 |
| TGFalpha | -1.08 | (-6.852, 4.693) | 0.7145 | 0.9732 |
| CLEC7A | 0.656 | (-2.853, 4.166) | 0.7145 | 0.9732 |
| CD5 | 1.191 | (-5.388, 7.771) | 0.7232 | 0.9732 |
| DCTN1 | 0.243 | (-1.139, 1.625) | 0.7307 | 0.9732 |
| FCRL3 | -0.676 | (-4.563, 3.211) | 0.7337 | 0.9732 |
| DNER | 1.408 | (-6.687, 9.502) | 0.7337 | 0.9732 |
| ITGA6 | -0.399 | (-2.838, 2.04) | 0.7488 | 0.9732 |
| HGF | -0.897 | (-6.812, 5.018) | 0.7667 | 0.9732 |
| IL7 | -0.425 | (-3.354, 2.504) | 0.7766 | 0.9732 |
| CSF1 | 1.569 | (-9.717, 12.855) | 0.7857 | 0.9732 |
| CST5 | -0.456 | (-3.796, 2.885) | 0.7896 | 0.9732 |
| CD244 | 0.556 | (-3.878, 4.99) | 0.8062 | 0.9732 |
| IRAK1 | 0.224 | (-1.588, 2.035) | 0.8090 | 0.9732 |
| PRDX5 | -0.158 | (-1.484, 1.168) | 0.8156 | 0.9732 |
| IL18R1 | -0.6 | (-5.846, 4.646) | 0.8229 | 0.9732 |
| LILRB4 | -0.49 | (-5.142, 4.162) | 0.8367 | 0.9732 |
| Flt3L | -0.459 | (-5.347, 4.428) | 0.8541 | 0.9732 |
| CLEC4G | 0.412 | (-4.093, 4.916) | 0.8581 | 0.9732 |
| BTN3A2 | 0.417 | (-4.178, 5.011) | 0.8592 | 0.9732 |
| HEXIM1 | 0.131 | (-1.323, 1.584) | 0.8604 | 0.9732 |
| CXCL11 | 0.19 | (-1.938, 2.318) | 0.8613 | 0.9732 |
| TNFB | -0.349 | (-4.399, 3.7) | 0.8660 | 0.9732 |
| DFFA | 0.193 | (-2.055, 2.442) | 0.8663 | 0.9732 |
| TNFSF14 | 0.269 | (-3.149, 3.687) | 0.8776 | 0.9732 |
| CCL25 | 0.256 | (-3.094, 3.607) | 0.8810 | 0.9732 |
| NTF4 | -0.269 | (-3.964, 3.427) | 0.8870 | 0.9732 |
| CXADR | -0.296 | (-4.464, 3.871) | 0.8893 | 0.9732 |
| BACH1 | 0.099 | (-1.428, 1.626) | 0.8993 | 0.9732 |
| DDX58 | -0.172 | (-2.946, 2.602) | 0.9033 | 0.9732 |
| CCL28 | 0.302 | (-4.839, 5.444) | 0.9084 | 0.9732 |
| CXCL9 | 0.127 | (-2.16, 2.414) | 0.9134 | 0.9732 |
| TPSAB1 | -0.128 | (-2.503, 2.247) | 0.9163 | 0.9732 |
| PPP1R9B | 0.077 | (-1.355, 1.508) | 0.9167 | 0.9732 |
| TRAIL | -0.293 | (-5.79, 5.204) | 0.9169 | 0.9732 |
| SRPK2 | 0.067 | (-1.233, 1.366) | 0.9197 | 0.9732 |
| **Variable** | **Estimates** | **(95% CI)** | **p-value** | **q-value** |
| MGMT | -0.054 | (-1.152, 1.044) | 0.9239 | 0.9732 |
| MMP10 | 0.118 | (-2.307, 2.543) | 0.9240 | 0.9732 |
| SPRY2 | -0.055 | (-1.192, 1.083) | 0.9252 | 0.9732 |
| HSD11B1 | 0.184 | (-3.999, 4.367) | 0.9315 | 0.9732 |
| PLXNA4 | 0.044 | (-0.986, 1.073) | 0.9336 | 0.9732 |
| FCRL6 | 0.103 | (-2.62, 2.825) | 0.9411 | 0.9732 |
| ST1A1 | 0.061 | (-1.644, 1.766) | 0.9443 | 0.9732 |
| ITM2A | -0.051 | (-2.126, 2.024) | 0.9616 | 0.9835 |
| CLEC4A | 0.071 | (-4.554, 4.696) | 0.9760 | 0.9886 |
| EIF4G1 | 0.012 | (-1.019, 1.043) | 0.9812 | 0.9886 |
| HCLS1 | 0.003 | (-1.472, 1.477) | 0.9974 | 0.9974 |

| **Table S2.** Associations between levels of plasma proteins and respiratory resistance (R_5_) as z-score analyzed by linear regression models adjusted for sex. P-values are uncorrected while q-values were derived by Benjamini Hochberg correction with false discovery rate 0.05. | | | | |
| --- | --- | --- | --- | --- |
| **Variable** | **Estimates** | **(95% CI)** | **p-value** | **q-value** |
| TNF | 0.638 | (0.294, 0.983) | 0.0004 | 0.0540 |
| CCL19 | 0.414 | (0.175, 0.654) | 0.0009 | 0.0624 |
| TREM1 | 0.616 | (0.053, 1.18) | 0.0348 | 0.9993 |
| uPA | 0.51 | (0.004, 1.015) | 0.0502 | 0.9993 |
| ITM2A | 0.219 | (0, 0.438) | 0.0528 | 0.9993 |
| PDL1 | 0.335 | (-0.012, 0.681) | 0.0603 | 0.9993 |
| NCR1 | 0.473 | (-0.022, 0.967) | 0.0646 | 0.9993 |
| IL10RB | 0.523 | (-0.052, 1.098) | 0.0767 | 0.9993 |
| DNER | -0.605 | (-1.301, 0.09) | 0.0903 | 0.9993 |
| CDCP1 | 0.427 | (-0.074, 0.929) | 0.0973 | 0.9993 |
| FCRL3 | -0.379 | (-0.823, 0.065) | 0.0977 | 0.9993 |
| CKAP4 | 0.409 | (-0.071, 0.889) | 0.0989 | 0.9993 |
| TRANCE | 0.228 | (-0.043, 0.499) | 0.1009 | 0.9993 |
| SLAMF1 | 0.381 | (-0.087, 0.85) | 0.1126 | 0.9993 |
| CX3CL1 | 0.338 | (-0.115, 0.792) | 0.1458 | 0.9993 |
| CXCL10 | 0.132 | (-0.046, 0.31) | 0.1471 | 0.9993 |
| IL12B | 0.25 | (-0.088. 0.588) | 0.1495 | 0.9993 |
| HNMT | 0.308 | (-0.109, 0.726) | 0.1517 | 0.9993 |
| MILR1 | 0.257 | (-0.118, 0.632) | 0.1831 | 0.9993 |
| IL10RA | -0.107 | (-0.269, 0.055) | 0.1991 | 0.9993 |
| CLEC4D | 0.243 | (-0.126, 0.611) | 0.2003 | 0.9993 |
| CCL11 | 0.258 | (-0.138, 0.654) | 0.2035 | 0.9993 |
| HSD11B1 | -0.31 | (-0.789, 0.169) | 0.2079 | 0.9993 |
| LIFR | 0.463 | (-0.264, 1.19) | 0.2139 | 0.9993 |
| LY75 | 0.338 | (-0.207, 0.884) | 0.2273 | 0.9993 |
| CD5 | 0.333 | (-0.274, 0.94) | 0.2839 | 0.9993 |
| CD83 | -0.311 | (-0.884, 0.261) | 0.2892 | 0.9993 |
| CXCL9 | 0.107 | (-0.091, 0.306) | 0.2906 | 0.9993 |
| IL10 | 0.152 | (-0.132, 0.436) | 0.2954 | 0.9993 |
| DCBLD2 | 0.311 | (-.0274, 0.895) | 0.3008 | 0.9993 |
| IFNgamma | 0.09 | (-0.085, 0.265) | 0.3167 | 0.9993 |
| KRT19 | -0.127 | (-0.38, 0.126) | 0.3267 | 0.9993 |
| IL18R1 | -0.223 | (-0.687, 0.24) | 0.3466 | 0.9993 |
| NT3 | 0.233 | (-0.257, 0.724) | 0.3529 | 0.9993 |
| TRAIL | 0.236 | (-0.268, 0.741) | 0.3603 | 0.9993 |
| LILRB4 | -0.248 | (-0.779, 0.284) | 0.3637 | 0.9993 |
| OSM | -0.09 | (-0.296, 0.115) | 0.3906 | 0.9993 |
| GDNF | -0.128 | (-0.429, 0.174) | 0.4071 | 0.9993 |
| **Variable** | **Estimates** | **(95% CI)** | **p-value** | **q-value** |
| CXADR | 0.217 | (-0.299, 0.734) | 0.4120 | 0.9993 |
| CSF1 | 0.442 | (-0.612, 1.496) | 0.4125 | 0.9993 |
| CXCL11 | 0.077 | (-0.108, 0.263) | 0.4150 | 0.9993 |
| CASP8 | -0.156 | (-0.53, 0.218) | 0.4157 | 0.9993 |
| TNFRSF9 | 0.148 | (-0.221, 0.517) | 0.4320 | 0.9993 |
| MCP4 | 0.103 | (-0.154, 0.361) | 0.4331 | 0.9993 |
| IL8 | -0.143 | (-0.503, 0.216) | 0.4364 | 0.9993 |
| CXCL5 | 0.044 | (-0.068, 0.156) | 0.4423 | 0.9993 |
| TNFB | 0.14 | (-0.218, 0.498) | 0.4442 | 0.9993 |
| VEGFA | 0.213 | (-0.352, 0.779) | 0.4605 | 0.9993 |
| CCL25 | 0.113 | (-0.19, 0.416) | 0.4660 | 0.9993 |
| ST1A1 | 0.055 | (-0.095, 0.204) | 0.4741 | 0.9993 |
| ENRAGE | -0.093 | (-0.352, 0.167) | 0.4857 | 0.9993 |
| DDX58 | -0.1 | (-0.385, 0.185) | 0.4943 | 0.9993 |
| CST5 | -0.104 | (-0.404, 0.197) | 0.5001 | 0.9993 |
| CNTNAP2 | 0.15 | (-0.291, 0.59) | 0.5071 | 0.9993 |
| FAM3B | 0.135 | (-0.278, 0.548) | 0.5240 | 0.9993 |
| CXCL6 | 0.075 | (-0.156, 0.306) | 0.5257 | 0.9993 |
| PTH1R | -0.143 | (-0.591, 0.304) | 0.5322 | 0.9993 |
| LAPTGFbeta1 | 0.131 | (-0.282, 0.543) | 0.5356 | 0.9993 |
| MMP1 | -0.055 | (-0.227, 0.118) | 0.5370 | 0.9993 |
| EBP1 | -0.061 | (-0.261, 0.139) | 0.5523 | 0.9993 |
| CLEC4G | 0.179 | (-0.424, 0.782) | 0.5631 | 0.9993 |
| CD244 | -0.112 | (-0.502, 0.279) | 0.5759 | 0.9993 |
| CLEC4C | -0.112 | (-0.513, 0.288) | 0.5844 | 0.9993 |
| TNFSF14 | -0.078 | (-0.372, 0.216) | 0.6039 | 0.9993 |
| IL6 | 0.068 | (-0.187, 0.322) | 0.6041 | 0.9993 |
| ITGB6 | 0.138 | (-0.39, 0.666) | 0.6107 | 0.9993 |
| FGF23 | 0.072 | (-0.206, 0.351) | 0.6115 | 0.9993 |
| IRAK1 | 0.047 | (-0.151, 0.245) | 0.6425 | 0.9993 |
| NTF4 | 0.102 | (-0.34, 0.543) | 0.6531 | 0.9993 |
| MCP1 | 0.096 | (-0.323, 0.514) | 0.6544 | 0.9993 |
| TWEAK | -0.119 | (-0.645, 0.407) | 0.6582 | 0.9993 |
| TPSAB1 | -0.064 | (-0.348, 0.221) | 0.6624 | 0.9993 |
| Flt3L | 0.095 | (-0.331, 0.521) | 0.6639 | 0.9993 |
| SRPK2 | 0.032 | (-0.113, 0.177) | 0.6655 | 0.9993 |
| GLB1 | -0.077 | (-0.434, 0.279) | 0.6718 | 0.9993 |
| SIT1 | 0.074 | (-0.268, 0.417) | 0.6726 | 0.9993 |
| LAMP3 | 0.088 | (-0.332, 0.508) | 0.6810 | 0.9993 |
| TGFalpha | 0.102 | (-0.399, 0.602) | 0.6914 | 0.9993 |
| CXCL1 | 0.033 | (-0.13 0.196) | 0.6950 | 0.9993 |
| CLEC4A | 0.102 | (-0.405, 0.609) | 0.6951 | 0.9993 |
| IFNLR1 | -0.105 | (-0.646, 0.436) | 0.7042 | 0.9993 |
| **Variable** | **Estimates** | **(95% C)** | **p-value** | **q-value** |
| SPRY2 | -0.024 | (-0.145, 0.098) | 0.7045 | 0.9993 |
| BACH1 | 0.032 | (-0.134, 0.199) | 0.7063 | 0.9993 |
| DFFA | 0.044 | (-0.193, 0.28) | 0.7192 | 0.9993 |
| DPP10 | 0.111 | (-0.493, 0.715) | 0.7193 | 0.9993 |
| MGMT | -0.021 | (-0.143, 0.101) | 0.7382 | 0.9993 |
| CD40 | 0.058 | (-0.282, 0.399) | 0.7382 | 0.9993 |
| LAG3 | -0.095 | (-0.668, 0.478) | 0.7454 | 0.9993 |
| KLRD1 | 0.062 | (-0.323, 0.447) | 0.7528 | 0.9993 |
| FGF19 | -0.025 | (-0.184, 0.134) | 0.7597 | 0.9993 |
| MCP2 | 0.035 | (-0.189, 0.259) | 0.7609 | 0.9993 |
| PRDX1 | 0.04 | (-0.221, 0.3) | 0.7664 | 0.9993 |
| PPP1R9B | 0.024 | (-0.132, 0.179) | 0.7674 | 0.9993 |
| MMP10 | -0.031 | (-0.242, 0.179) | 0.7713 | 0.9993 |
| CLEC6A | 0.054 | (-0.324, 0.431) | 0.7807 | 0.9993 |
| FCRL6 | -0.041 | (-0.333, 0.25) | 0.7819 | 0.9993 |
| TRIM21 | 0.035 | (-0.22, 0.291) | 0.7862 | 0.9993 |
| ITGA6 | 0.039 | (-0.255, 0.334) | 0.7938 | 0.9993 |
| CCL20 | 0.03 | (-0.198, 0.259) | 0.7959 | 0.9993 |
| HEXIM1 | 0.021 | (-0.139, 0.181) | 0.7987 | 0.9993 |
| STAMBP | -0.021 | (-0.189, 0.148) | 0.8096 | 0.9993 |
| ITGA11 | 0.046 | (-0.347, 0.438) | 0.8201 | 0.9993 |
| CD8A | 0.031 | (-0.258, 0.32) | 0.8338 | 0.9993 |
| OPG | 0.052 | (-0.458, 0.562) | 0.8428 | 0.9993 |
| MASP1 | -0.077 | (-0.836, 0.682) | 0.8434 | 0.9993 |
| IL17C | -0.02 | (-0.218, 0.179) | 0.8449 | 0.9993 |
| HGF | -0.052 | (-0.576, 0.472) | 0.8458 | 0.9993 |
| AXIN1 | 0.009 | (-0.099, 0.117) | 0.8670 | 0.9993 |
| CLEC7A | 0.032 | (-0.361, 0.426) | 0.8720 | 0.9993 |
| CD6 | -0.032 | (-0.423, 0.36) | 0.8745 | 0.9993 |
| PLXNA4 | 0.008 | (-0.1, 0.116) | 0.8863 | 0.9993 |
| IL18 | -0.026 | (-0.388, 0.335) | 0.8866 | 0.9993 |
| ADA | 0.033 | (-0.445, 0.511) | 0.8935 | 0.9993 |
| PRDX5 | -0.01 | (-0.15, 0.131) | 0.8941 | 0.9993 |
| BTN3A2 | -0.037 | (-0.586, 0.512) | 0.8952 | 0.9993 |
| DAPP1 | -0.006 | (-0.094, 0.082) | 0.8993 | 0.9993 |
| FGF2 | 0.012 | (-0.189, 0.214) | 0.9041 | 0.9993 |
| SH2B3 | 0.006 | (-0.103, 0.116) | 0.9108 | 0.9993 |
| CCL28 | 0.027 | (-0.463, 0.516) | 0.9154 | 0.9993 |
| SIRT2 | 0.007 | (-0.123, 0.137) | 0.9161 | 0.9993 |
| PIK3AP1 | -0.01 | (-0.204, 0.184) | 0.9175 | 0.9993 |
| IL15RA | 0.034 | (-0.613, 0.68) | 0.9185 | 0.9993 |
| IL7 | -0.012 | (-0.265, 0.24) | 0.9241 | 0.9993 |
| CCL4 | 0.013 | (-0.287, 0.313) | 0.9329 | 0.9993 |
| **Variable** | **Estimates** | **(95% C)** | **p-value** | **q-value** |
| CCL23 | -0.012 | (-0.327, 0.304) | 0.9424 | 0.9993 |
| SCF | 0.019 | (-0.513, 0.551) | 0.9445 | 0.9993 |
| EIF4G1 | -0.003 | (-0.112, 0.105) | 0.9529 | 0.9993 |
| IRF9 | 0.01 | (-0.314, 0.334) | 0.9532 | 0.9993 |
| STC1 | 0.014 | (-0.49, 0.518) | 0.9562 | 0.9993 |
| DCTN1 | 0.004 | (-0.15, 0.157) | 0.9625 | 0.9993 |
| FGF21 | 0.002 | (-0.119, 0.123) | 0.9711 | 0.9993 |
| NF2 | 0.002 | (-0.157, 0.16) | 0.9823 | 0.9993 |
| CCL3 | -0.002 | (-0.224, 0.219) | 0.9846 | 0.9993 |
| HCLS1 | 0.001 | (-0.157, 0.158) | 0.9925 | 0.9993 |
| AREG | 0 | (-0.285, 0.285) | 0.9993 | 0.9993 |

| **Table S3.** Associations between levels of plasma proteins and respiratory reactance (X_5_) as z-score analyzed by linear regression models adjusted for sex. P-values are uncorrected while q-values were derived by Benjamini Hochberg correction with false discovery rate 0.05. | | | | |
| --- | --- | --- | --- | --- |
| **Variable** | **Estimates** | **(95% CI)** | **p-value** | **q-value** |
| **CCL19** | **-0.533** | **(-0.818, -0.248)** | **0.0004** | **0.0483** |
| PTH1R | 0.526 | (0.042, 1.011) | 0.0360 | 0.9079 |
| BTN3A2 | 0.64 | (0.047, 1.233) | 0.0372 | 0.9079 |
| CXADR | 0.6 | (0.04, 1.16) | 0.0388 | 0.9079 |
| LIFR | -0.832 | (-1.696, 0.032) | 0.0612 | 0.9079 |
| FCRL3 | 0.438 | (-0.052, 0.929) | 0.0835 | 0.9079 |
| TPSAB1 | -0.271 | (-0.581, 0.04) | 0.0909 | 0.9079 |
| CDCP1 | -0.519 | (-1.119, 0.081) | 0.0923 | 0.9079 |
| IRF9 | 0.306 | (-0.047, 0.659) | 0.0926 | 0.9079 |
| CD83 | 0.538 | (-0.089. 1.166) | 0.0964 | 0.9079 |
| PPP1R9B | 0.139 | (-0.031, 0.308) | 0.1132 | 0.9079 |
| SH2B3 | 0.094 | (-0.025, 0.214) | 0.1262 | 0.9079 |
| FGF2 | 0.166 | (-0.054, 0.386) | 0.1428 | 0.9079 |
| CLEC4C | 0.331 | (-0.108, 0.769) | 0.1433 | 0.9079 |
| HCLS1 | 0.129 | (-0.043, 0.301) | 0.1464 | 0.9079 |
| IL10RA | 0.142 | (-0.051, 0.335) | 0.1526 | 0.9079 |
| CCL20 | -0.193 | (-0.465, 0.078) | 0.1650 | 0.9079 |
| PIK3AP1 | 0.15 | (-0.063, 0.363) | 0.1704 | 0.9079 |
| HEXIM1 | 0.123 | (-0.052, 0.298) | 0.1716 | 0.9079 |
| Flt3L | -0.346 | (-0.853, 0.161) | 0.1832 | 0.9079 |
| DAPP1 | 0.066 | (-0.031, 0.162) | 0.1871 | 0.9079 |
| SLAMF1 | -0.38 | (-0.941, 0.182) | 0.1877 | 0.9079 |
| IL8 | 0.288 | (-0.14, 0.717) | 0.1896 | 0.9079 |
| SPRY2 | 0.09 | (-0.043, 0.223) | 0.1896 | 0.9079 |
| TRAIL | 0.402 | (-0.2, 1.004) | 0.1928 | 0.9079 |
| uPA | -0.4 | (-1.01, 0.21) | 0.2009 | 0.9079 |
| ITGA6 | 0.202 | (-0.122, 0.525) | 0.2246 | 0.9079 |
| DDX58 | 0.194 | (-0.12, 0.508) | 0.2283 | 0.9079 |
| CKAP4 | 0.327 | (-0.209, 0.862) | 0.2352 | 0.9079 |
| DFFA | 0.157 | (-0.103, 0.417) | 0.2400 | 0.9079 |
| BACH1 | 0.108 | (-0.075, 0.291) | 0.2515 | 0.9079 |
| EIF4G1 | 0.07 | (-0.049, 0.19) | 0.2516 | 0.9079 |
| PLXNA4 | 0.069 | (-0.05, 0.188) | 0.2597 | 0.9079 |
| ST1A1 | -0.102 | (-0.28, 0.077) | 0.2662 | 0.9079 |
| PRDX5 | 0.088 | (-0.067, 0.243) | 0.2666 | 0.9079 |
| CLEC4A | -0.317 | (-0.875, 0.241) | 0.2682 | 0.9079 |
| DCTN1 | 0.095 | (-0.074, 0.263) | 0.2746 | 0.9079 |
| LILRB4 | 0.328 | (-0.26, 0.915) | 0.2773 | 0.9079 |
| PDL1 | -0.23 | (-0.648, 0.188) | 0.2823 | 0.9079 |
| **Variable** | **Estimates** | **(95% CI)** | **p-value** | **q-value** |
| VEGFA | -0.363 | (-1.038, 0.312) | 0.2938 | 0.9079 |
| SRPK2 | 0.086 | (-0.074, 0.246) | 0.2938 | 0.9079 |
| CXCL1 | 0.103 | (-0.092, 0.298) | 0.3021 | 0.9079 |
| NF2 | 0.092 | (-0.082, 0.266) | 0.3021 | 0.9079 |
| CD244 | -0.246 | (-0.712, 0.22) | 0.3028 | 0.9079 |
| MASP1 | 0.439 | (-0.396, 1.275) | 0.3055 | 0.9079 |
| OPG | -0.317 | (-0.925, 0.291) | 0.3093 | 0.9079 |
| MGMT | 0.068 | (-0.066, 0.202) | 0.3210 | 0.9117 |
| LAMP3 | 0.234 | (-0.229, 0.697) | 0.3254 | 0.9117 |
| TWEAK | -0.313 | (-0.941, 0.315) | 0.3309 | 0.9117 |
| PRDX1 | 0.139 | (-0.147, 0.426) | 0.3431 | 0.9176 |
| IRAK1 | 0.105 | (-0.113, 0.323) | 0.3467 | 0.9176 |
| LAPTGFbeta1 | -0.233 | (-0.726, 0.259) | 0.3546 | 0.9187 |
| TRANCE | -0.152 | (-0.478, 0.174) | 0.3612 | 0.9187 |
| HNMT | -0.215 | (-0.681, 0.251) | 0.3675 | 0.9187 |
| IL10RB | -0.312 | (-1.006, 0.382) | 0.3800 | 0.9301 |
| MCP4 | -0.135 | (-0.444, 0.173) | 0.3915 | 0.9301 |
| NTF4 | -0.214 | (-0.701, 0.274) | 0.3927 | 0.9301 |
| CD6 | -0.194 | (-0.661, 0.273) | 0.4160 | 0.9682 |
| CX3CL1 | -0.223 | (-0.769, 0.322) | 0.4236 | 0.9692 |
| MCP2 | 0.096 | (-0.172, 0.364) | 0.4829 | 0.9947 |
| KRT19 | 0.095 | (-0.186, 0.376) | 0.5076 | 0.9947 |
| NCR1 | -0.187 | (-0.744, 0.37) | 0.5125 | 0.9947 |
| LAG3 | 0.212 | (-0.422, 0.845) | 0.5145 | 0.9947 |
| CNTNAP2 | 0.162 | (-0.326, 0.649) | 0.5169 | 0.9947 |
| CD40 | -0.128 | (-0.536, 0.279) | 0.5380 | 0.9947 |
| CD5 | -0.229 | (-0.957, 0.499) | 0.5392 | 0.9947 |
| ITGB6 | -0.181 | (-0.766, 0.403) | 0.5446 | 0.9947 |
| MILR1 | -0.129 | (-0.548, 0.29) | 0.5485 | 0.9947 |
| AXIN1 | -0.039 | (-0.168, 0.09) | 0.5558 | 0.9947 |
| ITGA11 | -0.127 | (-0.56, 0.307) | 0.5687 | 0.9947 |
| IL12B | -0.117 | (-0.524, 0.291) | 0.5757 | 0.9947 |
| EBP1 | 0.068 | (-0.172, 0.308) | 0.5785 | 0.9947 |
| OSM | 0.069 | (-0.177, 0.316) | 0.5823 | 0.9947 |
| CCL28 | -0.163 | (-0.748, 0.423) | 0.5870 | 0.9947 |
| CLEC4D | 0.114 | (-0.297, 0.525) | 0.5887 | 0.9947 |
| SIT1 | -0.104 | (-0.483, 0.275) | 0.5917 | 0.9947 |
| ITM2A | -0.066 | (-0.313, 0.181) | 0.6025 | 0.9947 |
| FAM3B | 0.119 | (-0.339, 0.577) | 0.6129 | 0.9947 |
| AREG | 0.081 | (-0.234, 0.397) | 0.6142 | 0.9947 |
| CLEC7A | 0.108 | (-0.327, 0.543) | 0.6278 | 0.9947 |
| CSF1 | -0.307 | (-1.57, 0.956) | 0.6347 | 0.9947 |
| CXCL10 | -0.05 | (-0.264, 0.165) | 0.6515 | 0.9947 |
| **Variable** | **Estimates** | **(95% CI)** | **p-value** | **q-value** |
| CCL25 | -0.084 | (-0.4470 0.279) | 0.6517 | 0.9947 |
| IL18 | -0.094 | (-0.527, 0.338) | 0.6700 | 0.9947 |
| FGF21 | -0.031 | (-0.176, 0.113) | 0.6732 | 0.9947 |
| TNF | -0.092 | (-0.523, 0.34) | 0.6784 | 0.9947 |
| LY75 | 0.129 | (-0.48, 0.737) | 0.6797 | 0.9947 |
| TRIM21 | 0.059 | (-0.224, 0.342) | 0.6849 | 0.9947 |
| HSD11B1 | -0.111 | (-0.645, 0.424) | 0.6861 | 0.9947 |
| CXCL11 | 0.046 | (-0.177, 0.269) | 0.6866 | 0.9947 |
| IFNgamma | 0.043 | (-0.168, 0.253) | 0.6921 | 0.9947 |
| MMP1 | -0.041 | (-0.248, 0.166) | 0.6958 | 0.9947 |
| ENRAGE | 0.059 | (-0.252, 0.37) | 0.7125 | 0.9947 |
| FCRL6 | 0.06 | (-0.263, 0.383) | 0.7168 | 0.9947 |
| HGF | 0.115 | (-0.512, 0.742) | 0.7208 | 0.9947 |
| IFNLR1 | 0.106 | (-0.493, 0.705) | 0.7298 | 0.9947 |
| IL6 | -0.053 | (-0.358, 0.251) | 0.7316 | 0.9947 |
| ADA | -0.099 | (-0.671, 0.473) | 0.7350 | 0.9947 |
| TNFRSF9 | 0.074 | (-0.368, 0.516) | 0.7428 | 0.9947 |
| DCBLD2 | 0.107 | (-0.544, 0.758) | 0.7485 | 0.9947 |
| STC1 | -0.086 | (-0.644, 0.472) | 0.7636 | 0.9947 |
| IL18R1 | -0.083 | (-0.639, 0.473) | 0.7696 | 0.9947 |
| GDNF | 0.051 | (-0.311, 0.413) | 0.7825 | 0.9947 |
| CLEC6A | 0.058 | (-0.36, 0.476) | 0.7855 | 0.9947 |
| MMP10 | -0.034 | (-0.286, 0.218) | 0.7899 | 0.9947 |
| CLEC4G | -0.088 | (-0.757. 0.581) | 0.7969 | 0.9947 |
| TREM1 | -0.075 | (-0.715, 0.565) | 0.8196 | 0.9947 |
| TNFB | -0.049 | (-0.478, 0.38) | 0.8233 | 0.9947 |
| CD8A | 0.038 | (-0.308, 0.384) | 0.8301 | 0.9947 |
| CST5 | 0.039 | (-0.321, 0.399) | 0.8324 | 0.9947 |
| GLB1 | -0.036 | (-0.432, 0.359) | 0.8572 | 0.9947 |
| IL15RA | 0.068 | (-0.705, 0.842) | 0.8632 | 0.9947 |
| MCP1 | 0.042 | (-0.459, 0.544) | 0.8684 | 0.9947 |
| FGF19 | 0.016 | (-0.174, 0.206) | 0.8687 | 0.9947 |
| CXCL6 | 0.023 | (-0.254, 0.299) | 0.8726 | 0.9947 |
| CCL3 | 0.021 | (-0.244, 0.286) | 0.8782 | 0.9947 |
| CCL4 | 0.028 | (-0.331, 0.387) | 0.8797 | 0.9947 |
| CCL11 | -0.034 | (-0.511, 0.443) | 0.8892 | 0.9947 |
| IL17C | -0.016 | (-0.253, 0.221) | 0.8952 | 0.9947 |
| KLRD1 | -0.029 | (-0.456, 0.398) | 0.8957 | 0.9947 |
| FGF23 | 0.022 | (-0.311, 0.356) | 0.8959 | 0.9947 |
| STAMBP | -0.013 | (-0.215, 0.189) | 0.8989 | 0.9947 |
| SCF | -0.035 | (-0.671, 0.601) | 0.9142 | 0.9953 |
| CXCL9 | 0.012 | (-0.227, 0.25) | 0.9229 | 0.9953 |
| SIRT2 | -0.008 | (-0.164, 0.148) | 0.9236 | 0.9953 |
| **Variable** | **Estimates** | **(95% CI)** | **p-value** | **q-value** |
| CASP8 | -0.02 | (-0.469, 0.429) | 0.9318 | 0.9953 |
| TNFSF14 | -0.011 | (-0.364, 0.341) | 0.9496 | 0.9953 |
| CCL23 | 0.009 | (-0.369, 0.386) | 0.9641 | 0.9953 |
| DPP10 | -0.014 | (-0.684, 0.655) | 0.9665 | 0.9953 |
| NT3 | -0.01 | (-0.599, 0.579) | 0.9735 | 0.9953 |
| IL10 | 0.004 | (-0.338, 0.345) | 0.9824 | 0.9953 |
| TGFalpha | 0.007 | (-0.593, 0.606) | 0.9826 | 0.9953 |
| CXCL5 | 0.001 | (-0.133, 0.136) | 0.9829 | 0.9953 |
| DNER | 0.003 | (-0.838, 0.844) | 0.9945 | 0.9953 |
| IL7 | -0.001 | (-0.303, 0.301) | 0.9953 | 0.9953 |
