# Supplementary material for: Systemic inflammatory biomarkers in relation to lung function and exercise‐induced bronchoconstriction in adolescents
Source: Pediatr Allergy Immunol. 2025 Oct 24;36(10):e70231. doi: 10.1111/pai.70231 (PMC12550650; doi:10.1111/pai.70231)
Supplement: Supplementary file 1 — Table S1. [file PAI-36-e70231-s001.zip › pai70231-sup-0002-TableS4-S6@Supplement_airway_respone_exercise.docx]

| **Table S4.** Associations between levels of plasma proteins and ΔFEV_1_ analyzed by linear regression models adjusted for sex. P-values are uncorrected while q-values were derived by Benjamini Hochberg correction with false discovery rate 0.05. | | | | |
| --- | --- | --- | --- | --- |
| **Variable** | **Estimates** | **(95% CI)** | **p-value** | **q-value** |
| FGF21 | -1.376 | (-0.322, -2.43) | 0.0116 | 0.9668 |
| IL15RA | -5.998 | (-0.282, -11.713) | 0.0416 | 0.9668 |
| CD8A | -2.589 | (-0.082, -5.097) | 0.0449 | 0.9668 |
| uPA | 4.496 | (-9.005, 0.013) | 0.0527 | 0.9668 |
| CLEC7A | 2.907 | (-6.003, 0.188) | 0.0677 | 0.9668 |
| CD83 | 3.825 | (-8.136, 0.485) | 0.0842 | 0.9668 |
| NT3 | 3.823 | (-8.178, 0.532) | 0.0876 | 0.9668 |
| PRDX1 | -1.969 | (-0.289, 4.227) | 0.0897 | 0.9668 |
| ST1A1 | -1.04 | (-0.284, 2.364) | 0.1258 | 0.9668 |
| MCP1 | 2.908 | (-6.611, 0.795) | 0.1260 | 0.9668 |
| TGFalpha | -3.376 | (-1.056, 7.807) | 0.1377 | 0.9668 |
| FGF23 | -1.7 | (-0.571, 3.971) | 0.1446 | 0.9668 |
| FCRL6 | 1.813 | (-4.241, 0.616) | 0.1457 | 0.9668 |
| OPG | 3.284 | (-7.82, 1.252) | 0.1581 | 0.9668 |
| ADA | 2.855 | (-7.113, 1.402) | 0.1909 | 0.9668 |
| CXCL10 | -1.074 | (-0.529, 2.677) | 0.1912 | 0.9668 |
| ITM2A | -1.2 | (-0.658, 3.057) | 0.2077 | 0.9668 |
| CXCL6 | -1.322 | (-0.732, 3.376) | 0.2092 | 0.9668 |
| TNFB | 2.039 | (-5.228, 1.151) | 0.2124 | 0.9668 |
| IL10RA | 0.86 | (-2.297, 0.576) | 0.2425 | 0.9668 |
| CCL19 | 1.3 | (-3.49, 0.89) | 0.2465 | 0.9668 |
| DCBLD2 | 2.516 | (-7.028, 1.996) | 0.2764 | 0.9668 |
| FGF19 | -0.764 | (-0.653, 2.182) | 0.2923 | 0.9668 |
| Flt3L | 2.051 | (-5.877, 1.775) | 0.2952 | 0.9668 |
| IL7 | -1.178 | (-1.078, 3.434) | 0.3080 | 0.9668 |
| TRANCE | -1.249 | (-1.184, 3.681) | 0.3161 | 0.9668 |
| BTN3A2 | -2.057 | (-2.075, 6.188) | 0.3309 | 0.9668 |
| GDNF | 1.308 | (-4.019, 1.404) | 0.3462 | 0.9668 |
| CXADR | 1.81 | (-5.601, 1.98) | 0.3508 | 0.9668 |
| IL10RB | -2.423 | (-2.723, 7.569) | 0.3577 | 0.9668 |
| PIK3AP1 | -0.747 | (-0.874, 2.369) | 0.3679 | 0.9668 |
| SIT1 | 1.404 | (-4.501, 1.693) | 0.3758 | 0.9668 |
| ITGB6 | -1.91 | (-2.544, 6.363) | 0.4021 | 0.9668 |
| TRIM21 | -0.855 | (-1.14, 2.849) | 0.4023 | 0.9668 |
| IFNgamma | 0.673 | (-2.251, 0.905) | 0.4045 | 0.9668 |
| MMP10 | -0.788 | (-1.091, 2.668) | 0.4124 | 0.9668 |
| CST5 | 1.1 | (-3.768, 1.567) | 0.4202 | 0.9668 |
| TPSAB1 | -0.862 | (-1.263, 2.986) | 0.4279 | 0.9668 |
| **Variable** | **Estimates** | **(95% CI)** | **p-value** | **q-value** |
| MMP1 | 0.606 | (-2.131, 0.918) | 0.4370 | 0.9668 |
| DCTN1 | -0.484 | (-0.742, 1.711) | 0.4402 | 0.9668 |
| CLEC4G | 1.581 | (-5.599, 2.438) | 0.4421 | 0.9668 |
| CD244 | -1.329 | (-2.124, 4.783) | 0.4518 | 0.9668 |
| IL8 | 1.241 | (-4.48, 1.999) | 0.4542 | 0.9668 |
| HNMT | 1.044 | (-3.808, 1.719) | 0.4601 | 0.9668 |
| NTF4 | -1.237 | (-2.07, 4.545) | 0.4646 | 0.9668 |
| IFNLR1 | 1.65 | (-6.102, 2.802) | 0.4688 | 0.9668 |
| CCL28 | -1.595 | (-2.795, 5.985) | 0.4775 | 0.9668 |
| CXCL1 | -0.531 | (-.931, 1.992) | 0.4778 | 0.9668 |
| DDX58 | -0.905 | (-1.591, 3.4) | 0.4785 | 0.9668 |
| MASP1 | 1.948 | (-7.372, 3.476) | 0.4827 | 0.9668 |
| MILR1 | -1.074 | (-1.918, 4.065) | 0.4830 | 0.9668 |
| CXCL9 | 0.636 | (-2.421, 1.148) | 0.4857 | 0.9668 |
| DPP10 | 1.561 | (-6.05, 2.929) | 0.4967 | 0.9668 |
| FGF2 | -0.544 | (-1.076, 2.164) | 0.5118 | 0.9668 |
| CLEC4A | -1.357 | (-2.772, 5.486) | 0.5205 | 0.9668 |
| SLAMF1 | -1.369 | (-2.878, 5.617) | 0.5285 | 0.9668 |
| KLRD1 | 0.964 | (-4.134, 2.206) | 0.5522 | 0.9668 |
| EBP1 | -0.534 | (-1.262, 2.33) | 0.5608 | 0.9668 |
| CCL23 | -0.82 | (-1.993, 3.633) | 0.5687 | 0.9668 |
| TNFSF14 | -0.754 | (-1.895, 3.404) | 0.5776 | 0.9668 |
| HEXIM1 | -0.365 | (-.926, 1.655) | 0.5806 | 0.9668 |
| FCRL3 | 0.98 | (-4.471, 2.511) | 0.5831 | 0.9668 |
| SH2B3 | -0.244 | (-0.644, 1.131) | 0.5917 | 0.9668 |
| AXIN1 | -0.257 | (-0.714, 1.227) | 0.6051 | 0.9668 |
| IL18 | 0.845 | (-4.078, 2.387) | 0.6090 | 0.9668 |
| SIRT2 | -0.305 | (-0.863, 1.473) | 0.6094 | 0.9668 |
| PPP1R9B | -0.328 | (-0.945, 1.6) | 0.6145 | 0.9668 |
| NF2 | -0.32 | (-0.931, 1.571) | 0.6166 | 0.9668 |
| IL6 | -0.585 | (-1.708, 2.878) | 0.6181 | 0.9668 |
| CLEC4C | -0.803 | (-2.524, 4.131) | 0.6367 | 0.9668 |
| SRPK2 | -0.276 | (-0.879, 1.43) | 0.6408 | 0.9668 |
| TRAIL | 1.05 | (-5.573, 3.474) | 0.6500 | 0.9668 |
| IRAK1 | -0.362 | (-1.248, 1.972) | 0.6602 | 0.9668 |
| LY75 | -0.969 | (-3.406, 5.345) | 0.6648 | 0.9668 |
| HCLS1 | -0.284 | (-1.026, 1.595) | 0.6711 | 0.9668 |
| LAPTGFbeta1 | -0.792 | (-2.917, 4.501) | 0.6762 | 0.9668 |
| MCP2 | -0.424 | (-1.594, 2.442) | 0.6812 | 0.9668 |
| PLXNA4 | -0.192 | (-0.725, 1.109) | 0.6822 | 0.9668 |
| IL10 | 0.531 | (-3.092, 2.03) | 0.6849 | 0.9668 |
| CDCP1 | -0.913 | (-3.635, 5.462) | 0.6946 | 0.9668 |
| ENRAGE | 0.467 | (-2.801, 1.866) | 0.6953 | 0.9668 |
| **Variable** | **Estimates** | **(95% CI)** | **p-value** | **q-value** |
| HSD11B1 | 0.767 | (-4.608, 3.075) | 0.6963 | 0.9668 |
| MCP4 | -0.457 | (-1.866, 2.779) | 0.7006 | 0.9668 |
| STC1 | 0.741 | (-4.539, 3.057) | 0.7026 | 0.9668 |
| NCR1 | 0.729 | (-4.475, 3.017) | 0.7034 | 0.9668 |
| PRDX5 | -0.226 | (-0.952, 1.405) | 0.7070 | 0.9668 |
| DAPP1 | -0.138 | (-0.595, 0.872) | 0.7122 | 0.9668 |
| FAM3B | -0.57 | (-2.489, 3.628) | 0.7156 | 0.9668 |
| IRF9 | -0.472 | (-2.107, 3.052) | 0.7202 | 0.9668 |
| AREG | 0.418 | (-2.816, 1.981) | 0.7334 | 0.9668 |
| TREM1 | 0.687 | (-4.711, 3.338) | 0.7385 | 0.9668 |
| CCL20 | 0.334 | (-2.382, 1.714) | 0.7496 | 0.9668 |
| CLEC4D | -0.456 | (-2.34, 3.252) | 0.7498 | 0.9668 |
| CD6 | 0.564 | (-4.085, 2.956) | 0.7539 | 0.9668 |
| BACH1 | -0.213 | (-1.145, 1.571) | 0.7590 | 0.9668 |
| SPRY2 | -0.156 | (-0.858, 1.171) | 0.7629 | 0.9668 |
| IL18R1 | 0.598 | (-4.781, 3.585) | 0.7797 | 0.9668 |
| SCF | -0.632 | (-4.063, 5.328) | 0.7922 | 0.9668 |
| CD5 | 0.707 | (-6.115, 4.701) | 0.7983 | 0.9668 |
| CXCL5 | -0.125 | (-0.887, 1.137) | 0.8088 | 0.9668 |
| CCL11 | -0.417 | (-3.171, 4.004) | 0.8203 | 0.9668 |
| IL17C | 0.193 | (-1.971, 1.585) | 0.8316 | 0.9668 |
| EIF4G1 | -0.094 | (-0.824, 1.011) | 0.8418 | 0.9668 |
| CD40 | -0.31 | (-2.739, 3.36) | 0.8421 | 0.9668 |
| ITGA6 | -0.208 | (-1.972, 2.388) | 0.8516 | 0.9668 |
| KRT19 | -0.182 | (-1.745, 2.11) | 0.8531 | 0.9668 |
| LAMP3 | 0.301 | (-3.736, 3.134) | 0.8639 | 0.9668 |
| CLEC6A | -0.27 | (-2.857, 3.397) | 0.8659 | 0.9668 |
| OSM | 0.16 | (-2.013, 1.694) | 0.8661 | 0.9668 |
| CASP8 | -0.256 | (-3.095, 3.606) | 0.8814 | 0.9668 |
| PDL1 | -0.236 | (-2.92, 3.392) | 0.8837 | 0.9668 |
| CX3CL1 | 0.298 | (-4.342, 3.747) | 0.8856 | 0.9668 |
| CXCL11 | 0.121 | (-1.786, 1.544) | 0.8867 | 0.9668 |
| TNF | 0.234 | (-3.465, 2.996) | 0.8872 | 0.9668 |
| DNER | 0.442 | (-6.729, 5.844) | 0.8905 | 0.9668 |
| CSF1 | 0.653 | (-9.94, 8.635) | 0.8907 | 0.9668 |
| CKAP4 | 0.256 | (-3.949, 3.438) | 0.8923 | 0.9668 |
| IL12B | -0.209 | (-2.837, 3.255) | 0.8930 | 0.9668 |
| CNTNAP2 | 0.242 | (-3.794, 3.309) | 0.8937 | 0.9668 |
| CCL25 | 0.18 | (-2.884, 2.524) | 0.8964 | 0.9668 |
| VEGFA | -0.329 | (-4.769, 5.427) | 0.8995 | 0.9668 |
| PTH1R | 0.251 | (-4.163, 3.66) | 0.8999 | 0.9668 |
| DFFA | -0.119 | (-1.881, 2.119) | 0.9073 | 0.9668 |
| CCL3 | -0.114 | (-1.875, 2.102) | 0.9109 | 0.9668 |
| **Variable** | **Estimates** | **(95% CI)** | **p-value** | **q-value** |
| GLB1 | -0.153 | (-2.662, 2.968) | 0.9154 | 0.9668 |
| MGMT | -0.044 | (-0.935, 1.022) | 0.9302 | 0.9668 |
| LAG3 | -0.176 | (-4.377, 4.729) | 0.9396 | 0.9668 |
| TNFRSF9 | 0.121 | (-3.332, 3.09) | 0.9412 | 0.9668 |
| ITGA11 | 0.106 | (-3.138, 2.926) | 0.9456 | 0.9668 |
| HGF | -0.161 | (-4.546, 4.868) | 0.9466 | 0.9668 |
| LILRB4 | -0.139 | (-4.02, 4.297) | 0.9480 | 0.9668 |
| LIFR | 0.208 | (-6.787, 6.371) | 0.9506 | 0.9668 |
| CCL4 | -0.082 | (-2.595, 2.759) | 0.9525 | 0.9668 |
| STAMBP | -0.038 | (-1.474, 1.549) | 0.9612 | 0.9684 |
| TWEAK | 0.054 | (-4.785, 4.677) | 0.9821 | 0.9821 |

| **Table S5.** Associations between levels of plasma proteins and ΔR_5_ analyzed by linear regression models adjusted for sex. P-values are uncorrected while q-values were derived by Benjamini Hochberg correction with false discovery rate 0.05. | | | | |
| --- | --- | --- | --- | --- |
| **Variable** | **Estimates** | **(95% CI)** | **p-value** | **q-value** |
| IL15RA | 32.707 | (13.323, 52.091) | 0.0012 | 0.1634 |
| IL10RB | 23.936 | (6.133, 41.739) | 0.0094 | 0.6345 |
| GLB1 | -10.542 | (-19.54, -1.543) | 0.0233 | 0.6534 |
| DPP10 | -17.102 | (-31.759, -2.446) | 0.0238 | 0.6534 |
| FGF19 | 5.7 | (0.8, 10.601) | 0.0242 | 0.6534 |
| ITGB6 | -15.719 | (-29.674, -1.764) | 0.0291 | 0.6538 |
| CLEC4C | -11.36 | (-22.209, -0.511) | 0.0422 | 0.6967 |
| STC1 | -12.584 | (-24.791, -0.377) | 0.0454 | 0.6967 |
| CCL19 | -7.85 | (-15.504, -0.195) | 0.0464 | 0.6967 |
| OPG | -15.538 | (-31.502, 0.425) | 0.0586 | 0.7905 |
| TRANCE | 7.374 | (-1.059, 15.806) | 0.0889 | 0.8859 |
| CLEC7A | 8.211 | (-1.744, 18.165) | 0.1084 | 0.8859 |
| ITM2A | -4.779 | (-10.673, 1.115) | 0.1145 | 0.8859 |
| NT3 | -12.227 | (-27.412, 2.959) | 0.1169 | 0.8859 |
| CCL11 | -9.865 | (-22.304, 2.574) | 0.1225 | 0.8859 |
| HGF | -13.165 | (-29.906, 3.576) | 0.1256 | 0.8859 |
| FAM3B | -7.854 | (-17.928, 2.221) | 0.1290 | 0.8859 |
| CXCL10 | 4.342 | (-1.348, 10.031) | 0.1371 | 0.8859 |
| TREM1 | -9.685 | (-22.495, 3.125) | 0.1408 | 0.8859 |
| IFNLR1 | -10.679 | (-24.819, 3.462) | 0.1413 | 0.8859 |
| MILR1 | -7.304 | (-17.082, 2.474) | 0.1457 | 0.8859 |
| TNFRSF9 | 8.566 | (-3.051, 20.183) | 0.1507 | 0.8859 |
| CASP8 | 10.329 | (-3.686, 24.344) | 0.1509 | 0.8859 |
| CLEC4A | -8.905 | (-21.833, 4.022) | 0.1794 | 0.9891 |
| CD244 | 9.444 | (-4.449, 23.336) | 0.1850 | 0.9891 |
| AREG | -4.805 | (-12.233, 2.623) | 0.2072 | 0.9891 |
| ENRAGE | 4.961 | (-3.086, 13.007) | 0.2291 | 0.9891 |
| TRAIL | 9.68 | (-6.076, 25.436) | 0.2306 | 0.9891 |
| NCR1 | -7.361 | (-19.38, 4.658) | 0.2322 | 0.9891 |
| IL18R1 | -8.756 | (-23.097, 5.586) | 0.2336 | 0.9891 |
| CXCL9 | 3.627 | (-2.576, 9.831) | 0.2538 | 0.9891 |
| LAG3 | -8.393 | (-22.775, 5.989) | 0.2548 | 0.9891 |
| FCRL6 | 4.244 | (-3.471, 11.959) | 0.2830 | 0.9891 |
| CD6 | 6.687 | (-5.612, 18.987) | 0.2885 | 0.9891 |
| CNTNAP2 | -6.285 | (-17.85, 5.289) | 0.2892 | 0.9891 |
| CSF1 | 17.443 | (-15.285, 50.17) | 0.2981 | 0.9891 |
| ADA | -8.231 | (-24.135, 7.672) | 0.3122 | 0.9891 |
| CLEC4D | -4.576 | (-13.428, 4.277) | 0.3130 | 0.9891 |
| LY75 | -7.234 | (-21.251, 6.784) | 0.3137 | 0.9891 |
| **Variable** | **Estimates** | **(95% CI)** | **p-value** | **q-value** |
| TGFalpha | 7.885 | (-7.656, 23.426) | 0.3218 | 0.9891 |
| PRDX1 | -3.7 | (-11.053, 3.653) | 0.3259 | 0.9891 |
| HSD11B1 | -6.129 | (-18.638, 6.38) | 0.3387 | 0.9891 |
| CCL23 | -4.838 | (-14.771, 5.094) | 0.3414 | 0.9891 |
| FGF23 | 4.119 | (-4.65, 12.888) | 0.3589 | 0.9891 |
| KLRD1 | 4.715 | (-5.63, 15.061) | 0.3734 | 0.9891 |
| CCL3 | 3.101 | (-3.797, 9.999) | 0.3799 | 0.9891 |
| TWEAK | 7.569 | (-9.782, 24.919) | 0.3941 | 0.9891 |
| CD5 | 8.3 | (-10.829, 27.429) | 0.3966 | 0.9891 |
| TPSAB1 | -3.111 | (-10.301, 4.078) | 0.3979 | 0.9891 |
| IL17C | -2.618 | (-8.868, 3.632) | 0.4131 | 0.9891 |
| CLEC6A | 4.083 | (-6.021, 14.187) | 0.4298 | 0.9891 |
| MCP4 | 3.382 | (-5.082, 11.847) | 0.4349 | 0.9891 |
| CXADR | 4.726 | (-7.297, 16.749) | 0.4425 | 0.9891 |
| MMP10 | 2.514 | (-4.061, 9.088) | 0.4550 | 0.9891 |
| IL7 | 3.095 | (-5.141, 11.331) | 0.4626 | 0.9891 |
| IL6 | 2.959 | (-4.958, 10.876) | 0.4652 | 0.9891 |
| IL18 | 4.186 | (-7.052, 15.425) | 0.4666 | 0.9891 |
| CD8A | 3.415 | (-5.945, 12.774) | 0.4758 | 0.9891 |
| MASP1 | -6.806 | (-25.469, 11.856) | 0.4760 | 0.9891 |
| NTF4 | -3.805 | (-14.252, 6.642) | 0.4766 | 0.9891 |
| CCL20 | -2.61 | (-9.798, 4.579) | 0.4780 | 0.9891 |
| CCL4 | 3.52 | (-6.225, 13.265) | 0.4802 | 0.9891 |
| PPP1R9B | 1.423 | (-2.738, 5.583) | 0.5039 | 0.9891 |
| IL12B | -3.589 | (-14.184, 7.005) | 0.5078 | 0.9891 |
| OSM | -2.127 | (-8.58, 4.326) | 0.5194 | 0.9891 |
| CST5 | 3.133 | (-6.451, 12.717) | 0.5228 | 0.9891 |
| MGMT | 1.034 | (-2.155, 4.222) | 0.5264 | 0.9891 |
| EIF4G1 | 0.972 | (-2.052, 3.997) | 0.5297 | 0.9891 |
| TNF | 3.503 | (-7.675, 14.682) | 0.5401 | 0.9891 |
| DCBLD2 | -4.392 | (-18.675, 9.891) | 0.5478 | 0.9891 |
| SCF | 5.006 | (-11.676, 21.688) | 0.5574 | 0.9891 |
| DAPP1 | 0.709 | (-1.698, 3.116) | 0.5648 | 0.9891 |
| MCP1 | -3.859 | (-17.04, 9.321) | 0.5670 | 0.9891 |
| CX3CL1 | 4.219 | (-10.275, 18.712) | 0.5693 | 0.9891 |
| BTN3A2 | -4.004 | (-17.883, 9.875) | 0.5728 | 0.9891 |
| CXCL1 | 1.441 | (-3.683, 6.565) | 0.5824 | 0.9891 |
| ITGA11 | -2.699 | (-12.306, 6.907) | 0.5828 | 0.9891 |
| VEGFA | 5.137 | (-13.273, 23.547) | 0.5854 | 0.9891 |
| MCP2 | 2.001 | (-5.301, 9.303) | 0.5921 | 0.9891 |
| CCL28 | 4.364 | (-11.599, 20.327) | 0.5930 | 0.9891 |
| PRDX5 | 1.035 | (-2.867, 4.937) | 0.6039 | 0.9891 |
| LILRB4 | -3.307 | (-16.691, 10.077) | 0.6290 | 0.9891 |
| **Variable** | **Estimates** | **(95% CI)** | **p-value** | **q-value** |
| CLEC4G | -2.999 | (-15.867, 9.869) | 0.6486 | 0.9891 |
| IL8 | 2.69 | (-9.142, 14.522) | 0.6566 | 0.9891 |
| TRIM21 | -1.511 | (-8.299, 5.278) | 0.6634 | 0.9891 |
| MMP1 | 1.215 | (-4.365, 6.795) | 0.6703 | 0.9891 |
| PTH1R | -3.062 | (-17.247, 11.122) | 0.6729 | 0.9891 |
| PLXNA4 | 0.643 | (-2.467, 3.753) | 0.6860 | 0.9891 |
| IRF9 | -1.782 | (-10.579, 7.014) | 0.6919 | 0.9891 |
| LIFR | 4.568 | (-18.218, 27.355) | 0.6950 | 0.9891 |
| CXCL5 | 0.713 | (-2.851, 4.277) | 0.6957 | 0.9891 |
| SRPK2 | 0.759 | (-3.102, 4.62) | 0.7007 | 0.9891 |
| HCLS1 | 0.82 | (-3.487, 5.126) | 0.7097 | 0.9891 |
| FGF2 | -1.053 | (-6.601, 4.496) | 0.7107 | 0.9891 |
| CXCL11 | 1.165 | (-5.019, 7.35) | 0.7124 | 0.9891 |
| SH2B3 | 0.558 | (-2.426, 3.542) | 0.7145 | 0.9891 |
| SPRY2 | 0.621 | (-2.72, 3.962) | 0.7162 | 0.9891 |
| DDX58 | -1.5 | (-9.624, 6.624) | 0.7180 | 0.9891 |
| KRT19 | 1.053 | (-5.066, 7.171) | 0.7364 | 0.9897 |
| EBP1 | 1.045 | (-5.64, 7.73) | 0.7598 | 0.9897 |
| uPA | -2.496 | (-18.601, 13.61) | 0.7618 | 0.9897 |
| ST1A1 | 0.691 | (-4.189, 5.572) | 0.7817 | 0.9897 |
| CD83 | 2.014 | (-12.217, 16.246) | 0.7819 | 0.9897 |
| IL10 | 1.236 | (-7.593, 10.066) | 0.7841 | 0.9897 |
| FCRL3 | 1.486 | (-9.809, 12.782) | 0.7969 | 0.9897 |
| ITGA6 | 1.015 | (-6.79, 8.82) | 0.7992 | 0.9897 |
| LAPTGFbeta1 | 1.873 | (-12.634, 16.379) | 0.8006 | 0.9897 |
| HEXIM1 | 0.55 | (-3.725, 4.825) | 0.8013 | 0.9897 |
| CCL25 | 1.077 | (-8.424, 10.579) | 0.8245 | 0.9897 |
| LAMP3 | -1.177 | (-11.962, 9.608) | 0.8310 | 0.9897 |
| DCTN1 | 0.43 | (-3.647, 4.507) | 0.8366 | 0.9897 |
| DFFA | -0.705 | (-7.683, 6.272) | 0.8432 | 0.9897 |
| CDCP1 | -1.552 | (-17.484, 14.381) | 0.8489 | 0.9897 |
| CKAP4 | 1.164 | (-11.003, 13.331) | 0.8516 | 0.9897 |
| CD40 | 0.94 | (-10.681, 12.561) | 0.8742 | 0.9897 |
| IFNgamma | -0.432 | (-5.902, 5.037) | 0.8771 | 0.9897 |
| SLAMF1 | 1.165 | (-14.456, 16.786) | 0.8840 | 0.9897 |
| Flt3L | -0.992 | (-14.405, 12.42) | 0.8849 | 0.9897 |
| STAMBP | 0.439 | (-5.5, 6.377) | 0.8851 | 0.9897 |
| TNFSF14 | 0.77 | (-9.729, 11.269) | 0.8859 | 0.9897 |
| DNER | -1.173 | (-22.914, 20.568) | 0.9159 | 0.9897 |
| SIRT2 | -0.229 | (-4.603, 4.145) | 0.9185 | 0.9897 |
| FGF21 | -0.187 | (-3.938, 3.563) | 0.9221 | 0.9897 |
| TNFB | -0.534 | (-11.701, 10.634) | 0.9255 | 0.9897 |
| PIK3AP1 | -0.231 | (-5.644, 5.183) | 0.9336 | 0.9897 |
| **Variable** | **Estimates** | **(95% CI)** | **p-value** | **q-value** |
| NF2 | 0.173 | (-4.017, 4.363) | 0.9356 | 0.9897 |
| AXIN1 | -0.148 | (-3.778, 3.483) | 0.9366 | 0.9897 |
| GDNF | -0.244 | (-9.618, 9.129) | 0.9593 | 0.9897 |
| BACH1 | 0.116 | (-4.419, 4.652) | 0.9600 | 0.9897 |
| IRAK1 | 0.117 | (-5.269, 5.504) | 0.9660 | 0.9897 |
| HNMT | 0.191 | (-9.211, 9.593) | 0.9683 | 0.9897 |
| PDL1 | 0.196 | (-10.897, 11.288) | 0.9725 | 0.9897 |
| IL10RA | -0.072 | (-5.376, 5.232) | 0.9789 | 0.9897 |
| CXCL6 | -0.087 | (-7.938, 7.764) | 0.9827 | 0.9897 |
| SIT1 | 0.067 | (-10.06, 10.193) | 0.9897 | 0.9897 |

| **Table S6.** Associations between levels of plasma proteins and ΔX_5_ analyzed by linear regression models adjusted for sex. Adjusted p-values were derived by Benjamini Hochberg correction with false discovery rate 0.05. P-values are uncorrected while q-values were derived by Benjamini Hochberg correction with false discovery rate 0.05. | | | | |
| --- | --- | --- | --- | --- |
| **Variable** | **Estimates** | **(95% CI)** | **p-value** | **q-value** |
| FGF19 | -0.137 | (-0.255. -0.02) | 0.0236 | 0.9788 |
| ITM2A | 0.153 | (0.01. 0.295) | 0.0375 | 0.9788 |
| IL15RA | -0.454 | (-0.935. 0.027) | 0.0665 | 0.9788 |
| TRAIL | -0.35 | (-0.726. 0.027) | 0.0712 | 0.9788 |
| AREG | 0.167 | (-0.014. 0.348) | 0.0728 | 0.9788 |
| CCL20 | 0.155 | (-0.015. 0.325) | 0.0769 | 0.9788 |
| ITGB6 | 0.297 | (-0.046. 0.641) | 0.0925 | 0.9788 |
| GDNF | 0.192 | (-0.034. 0.417) | 0.0978 | 0.9788 |
| MCP4 | -0.164 | (-0.357. 0.029) | 0.0978 | 0.9788 |
| GLB1 | 0.183 | (-0.037. 0.402) | 0.1051 | 0.9788 |
| TRANCE | -0.168 | (-0.372. 0.036) | 0.1083 | 0.9788 |
| CLEC7A | -0.2 | (-0.444. 0.045) | 0.1117 | 0.9788 |
| CCL19 | 0.139 | (-0.047. 0.326) | 0.1460 | 0.9788 |
| MCP2 | -0.124 | (-0.292. 0.043) | 0.1485 | 0.9788 |
| NT3 | 0.265 | (-0.103. 0.633) | 0.1606 | 0.9788 |
| CLEC4C | 0.191 | (-0.075. 0.458) | 0.1621 | 0.9788 |
| ADA | 0.25 | (-0.107. 0.608) | 0.1726 | 0.9788 |
| LAG3 | 0.242 | (-0.108. 0.592) | 0.1771 | 0.9788 |
| ENRAGE | -0.135 | (-0.329. 0.06) | 0.1772 | 0.9788 |
| CLEC4A | 0.212 | (-0.105. 0.53) | 0.1919 | 0.9788 |
| DPP10 | 0.236 | (-0.118. 0.591) | 0.1937 | 0.9788 |
| LILRB4 | 0.213 | (-0.108. 0.534) | 0.1956 | 0.9788 |
| OPG | 0.239 | (-0.143. 0.621) | 0.2223 | 0.9788 |
| IL10RB | -0.27 | (-0.705. 0.166) | 0.2269 | 0.9788 |
| FCRL3 | 0.164 | (-0.107. 0.436) | 0.2367 | 0.9788 |
| CLEC4D | 0.126 | (-0.086. 0.337) | 0.2458 | 0.9788 |
| MILR1 | 0.133 | (-0.1. 0.366) | 0.2649 | 0.9788 |
| CXCL10 | -0.076 | (-0.21. 0.059) | 0.2713 | 0.9788 |
| FGF23 | -0.118 | (-0.327. 0.092) | 0.2722 | 0.9788 |
| NCR1 | 0.164 | (-0.128. 0.456) | 0.2724 | 0.9788 |
| TRIM21 | 0.085 | (-0.068. 0.239) | 0.2788 | 0.9788 |
| TPSAB1 | 0.097 | (-0.081. 0.274) | 0.2876 | 0.9788 |
| BTN3A2 | 0.177 | (-0.154. 0.507) | 0.2964 | 0.9788 |
| PDL1 | 0.14 | (-0.123. 0.403) | 0.2983 | 0.9788 |
| CCL23 | 0.124 | (-0.113. 0.361) | 0.3077 | 0.9788 |
| PRDX1 | 0.087 | (-0.085. 0.26) | 0.3234 | 0.9788 |
| TNFB | 0.136 | (-0.133. 0.405) | 0.3236 | 0.9788 |
| IRF9 | 0.094 | (-0.102. 0.29) | 0.3483 | 0.9788 |
| STC1 | 0.141 | (-0.162. .0445) | 0.3623 | 0.9788 |
| **Variable** | **Estimates** | **(95% CI)** | **p-value** | **q-value** |
| IL17C | 0.069 | (-0.08. 0.218) | 0.3633 | 0.9788 |
| CXCL1 | -0.055 | (-0.178. 0.068) | 0.3802 | 0.9788 |
| FCRL6 | -0.08 | (-0.27. 0.11) | 0.4083 | 0.9788 |
| IL8 | -0.113 | (-0.384. 0.157) | 0.4133 | 0.9788 |
| SCF | -0.167 | (-0.566. 0.233) | 0.4151 | 0.9788 |
| CXCL5 | -0.035 | (-0.12. 0.049) | 0.4158 | 0.9788 |
| IL7 | -0.078 | (-0.268. 0.111) | 0.4199 | 0.9788 |
| PTH1R | 0.119 | (-0.181. 0.419) | 0.4376 | 0.9788 |
| HNMT | -0.085 | (-0.304. 0.133) | 0.4445 | 0.9788 |
| MMP10 | -0.059 | (-0.218. 0.099) | 0.4627 | 0.9788 |
| CKAP4 | 0.111 | (-0.189. 0.411) | 0.4702 | 0.9788 |
| IL10RA | 0.045 | (-0.078. 0.167) | 0.4733 | 0.9788 |
| HSD11B1 | 0.106 | (-0.202. 0.415) | 0.5010 | 0.9788 |
| CASP8 | -0.093 | (-0.375. 0.19) | 0.5210 | 0.9788 |
| ST1A1 | -0.037 | (-0.15. 0.076) | 0.5221 | 0.9788 |
| EIF4G1 | -0.024 | (-0.096. 0.049) | 0.5244 | 0.9788 |
| TGFalpha | -0.116 | (-0.493. 0.261) | 0.5466 | 0.9788 |
| TWEAK | -0.121 | (-0.517. 0.275) | 0.5500 | 0.9788 |
| IFNLR1 | 0.106 | (-0.244. 0.456) | 0.5533 | 0.9788 |
| PLXNA4 | -0.022 | (-0.096. 0.051) | 0.5570 | 0.9788 |
| SIT1 | 0.072 | (-0.17. 0.314) | 0.5611 | 0.9788 |
| CNTNAP2 | 0.084 | (-0.203. 0.37) | 0.5679 | 0.9788 |
| CX3CL1 | -0.1 | (-0.444. 0.244) | 0.5702 | 0.9788 |
| DFFA | 0.044 | (-0.109. 0.196) | 0.5736 | 0.9788 |
| EBP1 | -0.041 | (-0.192. 0.11) | 0.5974 | 0.9788 |
| CSF1 | -0.206 | (-1.001. 0.589) | 0.6116 | 0.9788 |
| DDX58 | 0.048 | (-0.142. 0.237) | 0.6242 | 0.9788 |
| MMP1 | 0.032 | (-0.098. 0.163) | 0.6258 | 0.9788 |
| CXCL6 | -0.043 | (-0.217. 0.131) | 0.6318 | 0.9788 |
| KLRD1 | -0.061 | (-0.309. 0.187) | 0.6320 | 0.9788 |
| CD5 | 0.11 | (-0.348. 0.569) | 0.6377 | 0.9788 |
| VEGFA | -0.102 | (-0.529. 0.324) | 0.6381 | 0.9788 |
| TREM1 | 0.075 | (-0.239. 0.39) | 0.6387 | 0.9788 |
| NTF4 | 0.069 | (-0.221. 0.359) | 0.6423 | 0.9788 |
| CCL28 | -0.087 | (-0.456. 0.281) | 0.6427 | 0.9788 |
| SPRY2 | -0.018 | (-0.096. 0.061) | 0.6604 | 0.9788 |
| uPA | 0.086 | (-0.3. 0.472) | 0.6636 | 0.9788 |
| CXCL9 | -0.032 | (-0.182. 0.118) | 0.6776 | 0.9788 |
| SLAMF1 | -0.075 | (-0.43. 0.281) | 0.6815 | 0.9788 |
| CCL25 | -0.047 | (-0.276. 0.181) | 0.6862 | 0.9788 |
| NF2 | 0.02 | (-0.078. 0.119) | 0.6877 | 0.9788 |
| DCBLD2 | 0.07 | (-0.274. 0.414) | 0.6909 | 0.9788 |
| CD6 | 0.059 | (-0.235. 0.354) | 0.6931 | 0.9788 |
| **Variable** | **Estimates** | **(95% CI)** | **p-value** | **q-value** |
| CXADR | 0.058 | (-0.232. 0.348) | 0.6957 | 0.9788 |
| IFNgamma | -0.026 | (-0.159. 0.106) | 0.6987 | 0.9788 |
| KRT19 | 0.029 | (-0.12. 0.178) | 0.7009 | 0.9788 |
| IL12B | -0.05 | (-0.307. 0.206) | 0.7023 | 0.9788 |
| IRAK1 | 0.024 | (-0.101. 0.15) | 0.7033 | 0.9788 |
| CCL3 | -0.032 | (-0.199. 0.135) | 0.7075 | 0.9788 |
| DNER | 0.099 | (-0.43. 0.628) | 0.7138 | 0.9788 |
| MGMT | -0.014 | (-0.09. 0.062) | 0.7180 | 0.9788 |
| CD40 | 0.046 | (-0.211. 0.302) | 0.7282 | 0.9788 |
| CLEC4G | -0.054 | (-0.365. 0.257) | 0.7344 | 0.9788 |
| PRDX5 | -0.016 | (-0.108. 0.076) | 0.7349 | 0.9788 |
| BACH1 | 0.018 | (-0.088. 0.125) | 0.7369 | 0.9788 |
| DAPP1 | -0.01 | (-0.068. 0.048) | 0.7385 | 0.9788 |
| CCL4 | -0.037 | (-0.263. 0.189) | 0.7469 | 0.9788 |
| Flt3L | 0.05 | (-0.271. 0.372) | 0.7584 | 0.9788 |
| CCL11 | -0.047 | (-0.347. 0.253) | 0.7596 | 0.9788 |
| STAMBP | 0.018 | (-0.109. 0.145) | 0.7772 | 0.9788 |
| TNF | -0.038 | (-0.31. 0.233) | 0.7828 | 0.9788 |
| FGF2 | -0.016 | (-0.143.0 .11) | 0.8002 | 0.9788 |
| FAM3B | 0.031 | (-0.212. 0.275) | 0.8007 | 0.9788 |
| TNFRSF9 | -0.035 | (-0.314. 0.243) | 0.8043 | 0.9788 |
| IL18 | 0.033 | (-0.24. 0.305) | 0.8146 | 0.9788 |
| IL6 | -0.023 | (-0.215. 0.169) | 0.8160 | 0.9788 |
| CXCL11 | 0.016 | (-0.125. 0.156) | 0.8270 | 0.9788 |
| CD8A | 0.024 | (-0.194. 0.242) | 0.8286 | 0.9788 |
| MCP1 | 0.031 | (-0.284. 0.347) | 0.8466 | 0.9788 |
| CD244 | -0.029 | (-0.323. 0.266) | 0.8484 | 0.9788 |
| IL10 | -0.021 | (-0.236. 0.194) | 0.8509 | 0.9788 |
| FGF21 | -0.008 | (-0.099. 0.083) | 0.8578 | 0.9788 |
| MASP1 | -0.039 | (-0.465. 0.387) | 0.8596 | 0.9788 |
| CD83 | -0.031 | (-0.373. 0.312) | 0.8608 | 0.9788 |
| ITGA6 | -0.015 | (-0.185. 0.156) | 0.8673 | 0.9788 |
| OSM | -0.013 | (-0.169. 0.142) | 0.8695 | 0.9788 |
| CLEC6A | -0.021 | (-0.27. 0.228) | 0.8701 | 0.9788 |
| LAPTGFbeta1 | -0.025 | (-0.336. 0.285) | 0.8730 | 0.9788 |
| SIRT2 | 0.007 | (-0.091. 0.105) | 0.8863 | 0.9788 |
| PIK3AP1 | 0.009 | (-0.118. 0.136) | 0.8919 | 0.9788 |
| LAMP3 | 0.017 | (-0.244. 0.278) | 0.8962 | 0.9788 |
| HGF | 0.025 | (-0.37. 0.42) | 0.9019 | 0.9788 |
| LY75 | 0.021 | (-0.319. 0.362) | 0.9023 | 0.9788 |
| PPP1R9B | -0.006 | (-0.107. 0.095) | 0.9082 | 0.9788 |
| SRPK2 | 0.005 | (-0.085. 0.095) | 0.9163 | 0.9788 |
| DCTN1 | 0.005 | (-0.092. 0.102) | 0.9213 | 0.9788 |
| **Variable** | **Estimates** | **(95% CI)** | **p-value** | **q-value** |
| SH2B3 | -0.003 | (-0.074. 0.068) | 0.9359 | 0.9788 |
| CDCP1 | 0.014 | (-0.368. 0.396) | 0.9425 | 0.9788 |
| AXIN1 | -0.003 | (-0.084. 0.079) | 0.9448 | 0.9788 |
| HEXIM1 | 0.003 | (-0.098. 0.105) | 0.9469 | 0.9788 |
| TNFSF14 | -0.007 | (-0.229. 0.215) | 0.9490 | 0.9788 |
| HCLS1 | -0.003 | (-0.105. 0.099) | 0.9500 | 0.9788 |
| LIFR | 0.009 | (-0.542. 0.559) | 0.9757 | 0.9977 |
| ITGA11 | -0.001 | (-0.237. 0.234) | 0.9914 | 1.0000 |
| CST5 | 0.001 | (-0.226. 0.227) | 0.9965 | 1.0000 |
| IL18R1 | 0 | (-0.35. 0.35) | 1.0000 | 1.0000 |
